# Ground State Destabilization by Anionic Nucleophiles Contributes to the Activity of Phosphoryl Transfer Enzymes

**DOI:** 10.1371/journal.pbio.1001599

**Published:** 2013-07-02

**Authors:** Logan D. Andrews, Tim D. Fenn, Daniel Herschlag

**Affiliations:** 1 Department of Chemical and Systems Biology, Stanford University, Stanford, California, United States of America; 2 Department of Bioengineering, Stanford University, Stanford, California, United States of America; 3 Department of Biochemistry, Stanford University, Stanford, California, United States of America; Brandeis University, United States of America

## Abstract

Enzymes stabilize transition states of reactions while limiting binding to ground states, as is generally required for any catalyst. Alkaline Phosphatase (AP) and other nonspecific phosphatases are some of Nature's most impressive catalysts, achieving preferential transition state over ground state stabilization of more than 10^22^-fold while utilizing interactions with only the five atoms attached to the transferred phosphorus. We tested a model that AP achieves a portion of this preference by destabilizing ground state binding via charge repulsion between the anionic active site nucleophile, Ser102, and the negatively charged phosphate monoester substrate. Removal of the Ser102 alkoxide by mutation to glycine or alanine increases the observed P_i_ affinity by orders of magnitude at pH 8.0. To allow precise and quantitative comparisons, the ionic form of bound P_i_ was determined from pH dependencies of the binding of P_i_ and tungstate, a P_i_ analog lacking titratable protons over the pH range of 5–11, and from the ^31^P chemical shift of bound P_i_. The results show that the P_i_ trianion binds with an exceptionally strong femtomolar affinity in the absence of Ser102, show that its binding is destabilized by ≥10^8^-fold by the Ser102 alkoxide, and provide direct evidence for ground state destabilization. Comparisons of X-ray crystal structures of AP with and without Ser102 reveal the same active site and P_i_ binding geometry upon removal of Ser102, suggesting that the destabilization does not result from a major structural rearrangement upon mutation of Ser102. Analogous P_i_ binding measurements with a protein tyrosine phosphatase suggest the generality of this ground state destabilization mechanism. Our results have uncovered an important contribution of anionic nucleophiles to phosphoryl transfer catalysis via ground state electrostatic destabilization and an enormous capacity of the AP active site for specific and strong recognition of the phosphoryl group in the transition state.

## Abstract 2

Enhanced phosphate binding by phosphatases upon removal of their anionic nucleophiles suggests that these enzymes use ground state destabilization by anionic active site nucleophiles as part of their catalytic arsenal.

## Author Summary

Enzymes use a variety of tools and strategies to enhance (catalyze) biological reactions; these include the use of general acids and bases, cofactors, and the employment of remote binding interactions to position substrates near reactive chemical groups. Phosphatases are some of Nature's best enzymes, affording exceptional rate enhancements to the biologically ubiquitous removal of a phosphate group from a substrate (dephosphorylation). The apparent challenge faced by nonspecific phosphatases is that their wide substrate specificity precludes the efficient use of remote binding interactions. Previous work suggested that phosphatases could use negatively charged chemical groups (anionic nucleophiles) at the active site to destabilize substrate binding without simultaneously destabilizing the transition state barrier—an elusive catalytic strategy known as preferential ground state destabilization. In this work, we test this ground state destabilization model of catalysis by removing the anionic active site nucleophile of alkaline phosphatase and observing the effects on the enzyme's affinity for a phosphate ligand. We find that alkaline phosphatase has an exceptionally strong affinity for phosphate, and provide clear evidence for ground state destabilization by the anionic active site nucleophile that, when present, forestalls substrate saturation and product inhibition, and enhances catalysis by at least a thousand fold.

## Introduction

Enzymes are central to biology, allowing chemical processes to be carried out rapidly and specifically. A range of enzymatic catalytic efficiencies of 10^6^–10^29^ fold have been observed [1],[2], with the more difficult chemical reactions generally exhibiting higher rate enhancements such that *k*_cat_/*K*_M_ values tend to cluster around 10^4^–10^5^ M^−1^ s^−1^ [3].

Decades of mechanistic enzymology have revealed several general strategies used by enzymes to achieve their prodigious rate enhancements, including the use of general acids and bases to facilitate proton transfers, coenzymes and metal cofactors to broaden the enzymatic reaction repertoire, and positioned hydrogen bond donors and acceptors and metal ions to stabilize rearranged charges in transition states. An additional hallmark of enzymes is the use of binding interactions with portions of their substrates that are not directly involved in the chemical transformation to position the reacting groups favorably for that transformation [4]–[14].

Nonspecific phosphatases, however, have little or no binding interactions with remote portions of the phosphate monoester substrates they hydrolyze, enabling them to liberate inorganic phosphate (P_i_) from any available monosubstituted phosphate source. Remarkably, these same phosphatases that do not use remote binding interactions for catalysis nonetheless exhibit some of the largest rate enhancements known. For example, alkaline phosphatase (AP) from *Escherichia coli* provides estimated rate enhancements of up to 10^27^-fold for the hydrolysis of a wide range of alkyl phosphates [15],[16]. According to transition state theory, this rate enhancement represents a stabilization energy of 37 kcal/mol 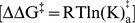 [16]. This energy, if expressed as binding energy in a ground state, would correspond to a dissociation constant of 10^−12^ fM, a trillion fold stronger than the affinity of avidin for biotin (*K*_d_≈1 fM, [17]).

**Figure 1:**
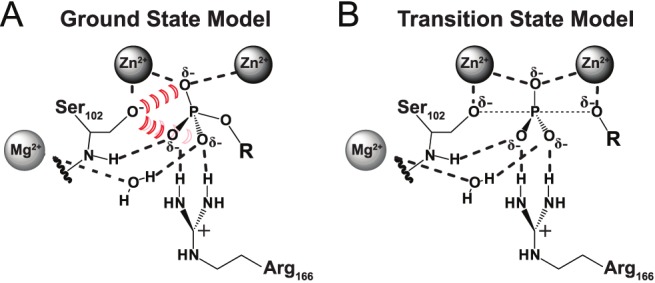
Active site models for the AP ground and transition state. AP ground state (A) and transition state (B) models based on previously solved X-ray crystal structures (PDB Codes 3TGO and 1B8J, respectively). Proposed active site contacts are illustrated with dashed lines. The proposed electrostatic destabilization from Ser102 in the ground state model is illustrated by the red hash marks.

Some of the interactions that contribute to AP's enormous transition state stabilization are readily assigned based on structural inspection, chemical insight, and functional studies (Figure 1B) [18],[19]. For example, in the transition state substantial negative charge builds up on the leaving group oxygen atom of the phosphoryl group such that the Zn^2+^ ion interacting with this group likely provides substantial stabilization [20]–[22]. Activation of the Ser102 nucleophile via Zn^2+^ coordination to give the serine alkoxide anion presumably also accelerates the enzyme-catalyzed reaction relative to the solution reaction that uses neutral water as the nucleophile [15], and further acceleration likely arises from positioning of the serine alkoxide nucleophile with respect to the reactive phosphoryl group within the active site.

Despite these recognizable strategies, the ability of AP to provide this enormous overall rate enhancement is not understood, and this rate enhancement is especially remarkable considering that, unlike in the avidin-biotin complex and in many enzymes that make extensive interactions with their entire substrates, the transition state interactions in AP appear to involve only five atoms—the oxygen atoms of the transferred phosphoryl group and of the incoming and outgoing groups (Figure 1B). These observations suggest that the AP active site could, in principle, provide exceptionally strong binding to simple phosphoryl compounds, a prediction that we test herein.

These considerations raise a further perplexing question that is also addressed herein. Transition state recognition involves the central phosphoryl group, a group also present in the ground state: How does AP distinguish so profoundly between its transition state and these same atoms in the ground state? The actual ground state affinity of AP for its substrate (*K*_d_>10 µM [20]; Figure 1A) is more than 10^22^-fold lower than the formal transition state affinity.

**Figure 2:**
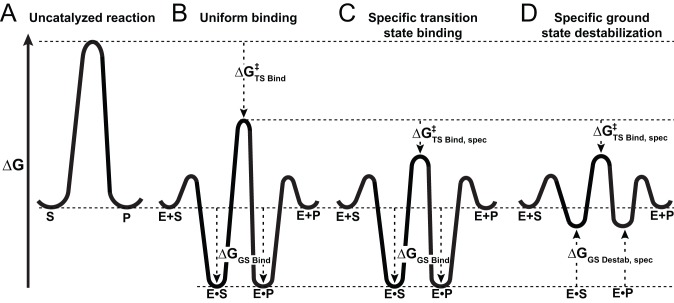
Free energy reaction profiles illustrating preferential E•S ground state destabilization. (A) Hypothetical uncatalyzed reaction profile. (B) Hypothetical enzyme that stabilizes the ground and transition states equally (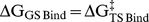) so that the resulting reaction barrier is equal to the uncatalyzed reaction barrier under saturating conditions. This enzyme is not a catalyst as stabilization of the transition state without parallel stabilization of the ground state is required for catalysis. (C) Hypothetical enzyme that makes additional, specific transition state stabilizing interactions, 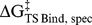 so that the reaction barrier between E.S and E.P is lower than that for the uncatalyzed barrier. This enzyme is a catalyst. (D) Hypothetical enzyme that makes specific ground state destabilizing interactions, 
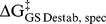
, to further enhance the catalytic properties of the enzyme relative to that in panel (C). This destabilization is discussed in the text.

Most generally, differential transition state versus ground state recognition is a requirement for any catalyst, as illustrated in Figure 2 (cf. A versus B). The simplest way to provide specific transition state stabilization (Figure 2C) is to introduce a group that can provide chemical catalysis—for example, the introduction of a base that can abstract a proton more efficiently than can water [23],[24]. A second common way to provide needed differential stabilization is to use binding interactions to position reacting groups within the active site, thereby entropically destabilizing the ground state (in terms of conformational entropy) but not equivalently destabilizing the transition state (Figure 2D), as these groups are, by definition, positioned with respect to one another in the transition state [4],[5],[25]–[27].

In addition to addressing the ability of AP to engage in remarkably strong ligand interactions, we provide evidence for electrostatic repulsion in AP that likely contributes to this critical discrimination against the ground state. We further show that there is analogous ground state destabilization in a structurally unrelated phosphatase that also contains an anionic nucleophile.

## Results and Discussion

### Model for Ground State Destabilization from the Ser102 Alkoxide Nucleophile

The AP active site contains three divalent metal ions and additional positively charged side chains in position to interact with the negatively charged phosphate monoester substrate (Figure 1A). The exception to this preponderance of positive charge is the active site nucleophile Ser102, which is Zn^2+^-coordinated and presumably negatively charged in the free enzyme with a p*K*_a_ of ≤5.5 [20]. Isotope-edited vibrational spectroscopy [16] and the pH dependence of binding of P_i_ and other ligands [20] indicated that when the P_i_ dianion 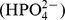 binds WT AP, its proton is lost to give bound P_i_ trianion 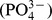, and a proton is taken up by a group on the enzyme so that there is no net proton loss to solution (Equation 1).
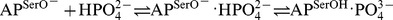
(1)


Based on the close positioning of the anionic Ser102, the negative charge of 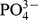, and the protonation of an enzymatic group upon P_i_ binding, we proposed Ser102 as the proton acceptor [16]. This model further predicts that the Ser102 anion substantially destabilizes binding of the phosphate ester dianion substrate in the ground state, as the substrate has no proton to transfer to Ser102 to eliminate the repulsion (Figure 1A). By limiting the stability of the E•S complex, ground state destabilization from Ser102 could prevent saturation at low substrate concentrations and reduce the barrier for reaction of bound substrate. This scenario is shown schematically in Figure 2D. A direct test of this proposal is that removal of Ser102 via mutagenesis should lead to stronger ground state binding, a test we carry out herein.

Ideally, to directly determine the effect of Ser102 on the E•S ground state stability, the affinity of a phosphate monoester would be compared in the presence and absence of Ser102. However, in the absence of Ser102, the affinity cannot be measured because trace P_i_ contamination in phosphate ester stocks (>0.1% as determined by ^31^P NMR) dominates binding due to the strong affinity of P_i_ (*vide infra*). In addition, trace contaminating phosphatase activity in the Ser102 mutant preparations generates P_i_ from any added phosphate ester (see Text S1). Thus, we turned to measurements of P_i_ affinities (the E•P_i_ ground state). Investigation of P_i_ interactions can provide a wealth of information, as P_i_ also serves as a substrate in an ^18^O-exchange reaction [28]–[32], several structures of P_i_-bound AP are available (e.g., [33]–[36]), its affinity is readily determined, and comparisons of the relative affinities of its di- and tri-anionic forms provide additional information.

### Observed Binding Affinity of P_i_ to Ser102 Mutants

To test if Ser102 destabilizes ground state binding, Ser102 was mutated to Gly or Ala, and the P_i_ binding affinity was compared to the P_i_ affinity of AP with Ser102 intact. We used a new ^32^P equilibrium-binding assay (see Materials and Methods) to measure the P_i_ affinity of the Ser102 AP mutants as the Ser102 mutants lack detectable activity, preventing the use of a kinetic inhibition assay to determine the P_i_ affinity that was previously used for WT AP and mutants with detectable activity ([20],[35],[37]; see Text S1). To test the validity and range of this assay we first determined the P_i_ affinity for WT AP. A 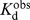 value of 0.26±0.07 µM was determined at pH 8.0 (Table 1; Text S2; Figure S1A), in reasonable agreement with values from prior kinetic inhibition assays (*K*_i_ = 0.5–1 µM) [16],[20],[38]. From a previous pH-dependent characterization of WT AP, the P_i_ affinity is expected to decrease as the pH is raised from 8.0 [20] and this result was also accurately reproduced with the equilibrium-binding assay (Figure S1D and E; see also Figure S1F and G). Controls carried out with mutant APs, described below and in the Supporting Information section, provide additional support for the accuracy of this assay.

**Table 1:** Binding of P_i_ to active site nucleophile mutants of AP.

Residue 102	Residue 166	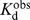 (nM)^a^	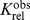 b
Ser	Arg	260±74	(1)
Gly	Arg	≤0.2	≥1,000
Ala	Arg	≤0.02	≥1×10^4^
Ser	Ser	(3.6±1.6)×10^5^	(1)
Gly	Ser	66±8	5,500
Ala	Ser	77±6	4,700

a The observed dissociation constants were determined at pH 8.0 in 100 mM NaMOPS, 100 mM NaCl, 1 mM MgCl_2_, and 100 µM ZnCl_2_ at 4°C.

b *K* 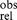 is the ratio of the observed P_i_ dissociation constant in the presence of Ser102 compared to the dissociation constant in the absence of Ser102: *K*_d_(Ser102)/*K*_d_(Gly or Ala102). Numbers greater than 1 represent stronger binding.

Mutation of Ser102 to either Gly or Ala led to binding of P_i_ that was so strong that, subsequent to uptake of ^32^P_i_, no significant dissociation of ^32^P_i_ bound to S102G or S102A AP could be observed following the addition of an excess of unlabeled P_i_ (see Materials and Methods), even after 100 h (Text S3; Figure S2A; see also Figure S3). These results provided upper limits for the dissociation rate constant, *k*_off_, of 2×10^−7^ s^−1^ for both S102G and S102A AP. This dissociation rate constant was too slow to allow equilibration prior to protein loss (presumably from irreversible denaturation) and thus prevented measurement of the P_i_ dissociation constants. Nevertheless, we could estimate the rate of uptake of ^32^P_i_ (Text S3 and figures therein) to obtain an upper limit for the equilibrium dissociation constant from this value and the above upper limit for *k*_off_ (Table 1; *K*_d_ = *k*_off_/*k*_on_). This dissociation constant is at least 10^3^-fold lower than that observed for P_i_ with WT AP, indicating stabilization of P_i_ binding upon removal of Ser102 (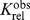; Table 1).

To reduce the P_i_ affinity of the Ser102 mutants to a measurable range, an additional mutation was introduced. Previous studies showed that mutation of Arg166, which interacts with two of the phosphoryl oxygen atoms (Figure 1), reduces P_i_ binding affinity by ∼10^3^-fold at pH 8.0 (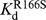 = 460 µM and 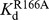 = 640 µM, [35],[39]; 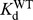 = 0.5–1 µM, [16],[20],[38]). P_i_ binding by the R166S AP single mutant could not be detected using the equilibrium-binding assay, as the concentrations of R166S AP needed to achieve binding in this assay are not readily obtained (Figure S7B). We therefore used the prior kinetic inhibition assay (Figure S7A) and repeated the P_i_ affinity measurement of R166S AP (Table 1) [35]. When Ser102 was mutated in the R166S AP background, P_i_ binding was observed using the equilibrium-binding assay (Text S4; Figure S8A and E) with dissociation constants of 66 and 77 nM for S102G/R166S and S102A/R166S AP, respectively, at pH 8.0 (Table 1). Measurements of the rate constants for P_i_ association and dissociation gave dissociation constants in reasonable agreement with the values determined in the equilibrium-binding assay (Text S4; Figure S8; Table S1). The mutations that remove Ser102 increase affinity by ∼10^3^-fold (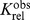; Table 1), providing additional strong support for a destabilizing influence of Ser102.

### Structural Analysis of P_i_ Bound to AP With and Without Ser102 Present

**Figure 3:**
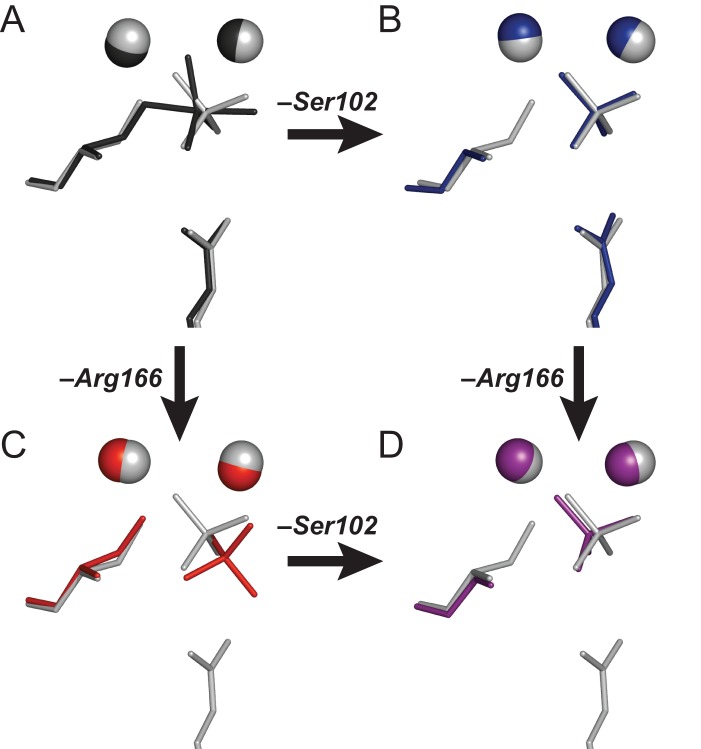
Structural comparisons of noncovalently bound P_i_ in AP and Ser102 mutants. (A) Overlay of WT AP with vanadate transition state analog covalently bound to Ser102 (black, 1PDB code 1B8J) and P_i_ noncovalently bound (gray, PDB code 3TGO). (B) Overlay of WT AP (grey) and S102G AP (blue, PDB code 1ELZ), both with bound P_i_. (C) Overlay of WT AP (grey) and R166S AP (red, PDB code 3CMR). Mutation of Arg166 to Ser results in rotation and 1.0 Å translation of the bound P_i_. (D) Overlay of WT AP (grey) and S102G/R166S AP (purple). Removal of the Arg166 side chain (R166S AP) results in a rearrangement of the bound P_i_ with Ser102 present (A→C) but not with Ser102 mutated (B→D) (see Text S5). While it is likely, based on the results herein and previously [16], that P_i_ is bound as the trianion in all cases and Ser102 is protonated when present, the X-ray data lack the resolution needed to identify protons.

The observed increase in P_i_ binding affinity of AP without Ser102 is expected to arise, according to our ground state destabilization model (Figure 1A), from the removal of the Ser102 negative charge. The Gly and Ala mutations give P_i_ binding affinities within 2-fold of one another, showing that the binding increase is not highly dependent on the steric properties of the group replacing the Ser102 side chain, but both side chain substitutions could allow bound P_i_ to rearrange to an alternative, more favorable, binding conformation. If this were the case, the weaker binding with Ser102 present could arise, at least in part, from steric hindrance rather than from electrostatic repulsion. However, the orientation of P_i_ bound to WT AP and the Ser102Gly and Ala mutants is indistinguishable (Figure 3B; [34]). The P_i_ binding geometry in the S102G/R166S AP double mutant (Figure 3D), solved herein (see Table S2 for refinement statistics), is also indistinguishable from that in WT AP. The structural analysis described in this section suggests that this alternative model does not hold and provides additional insights into active site features that contribute to alignment and positioning.

A comparison of our newly obtained S102G/R166S AP structure to a previously obtained structure of R166S AP [35] also reveals another active site property, an interplay between Ser102 and Arg166 in positioning bound P_i_ that underscores the role of Arg166 in specifically stabilizing the transition state. This interplay is depicted in Figure 3 and described in Text S5.

### Intrinsic Binding Affinities of Specific Ionic P_i_ Species to AP With and Without Ser102

The P_i_ binding results described above show that removing the Ser102 side chain increases the observed affinity by more than 10^3^-fold at pH 8.0. However, understanding the energetics of P_i_ binding and destabilization requires determination of equilibrium binding constants for individual P_i_ species to specified forms of WT and mutant APs. P_i_ has multiple ionic forms, whose relative populations depend on the solution pH (Equation 2) [40], and the form bound depends on these relative populations and the enzyme's binding preferences for each ionic form:
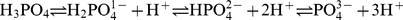
(2)


The following quantitative analyses reveal that removal of Ser102 unmasks an active site capable of very strong ground state binding of ∼1 fM and suggest a substantial role for Ser102 in destabilizing both substrate and product ground state binding by several orders of magnitude. Immediately following we describe the results underlying these conclusions, and their implications are addressed in the subsequent sections.

#### pH-dependent P_i_ binding to AP containing the Ser102 nucleophile

**Figure 4:**
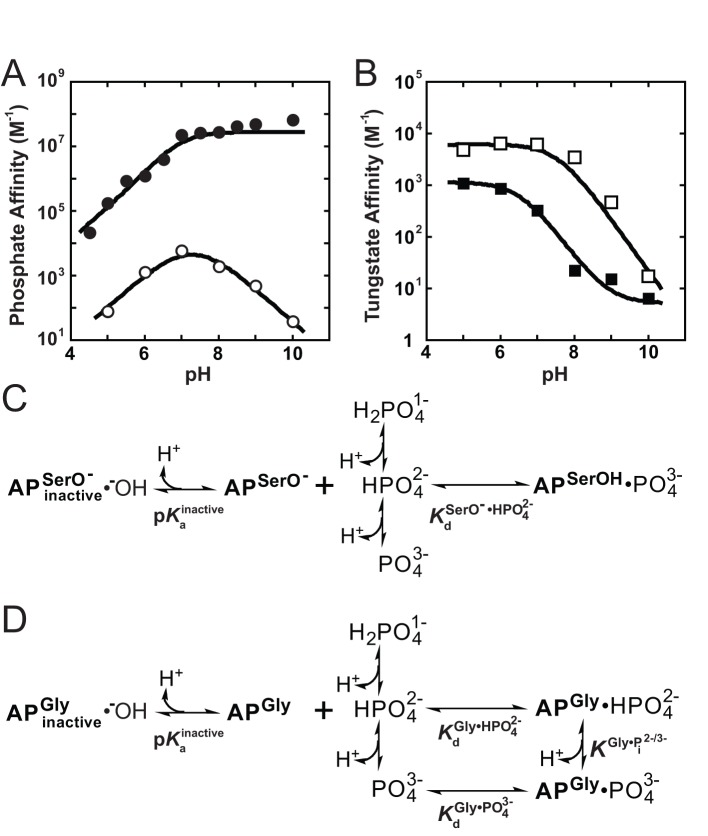
The pH dependence of P_i_ and tungstate binding for AP with and without Ser10 (A) The pH-dependent binding of P_i_ to R166S AP (open circles) and S102G/R166S AP (closed circles). See Methods for assay details. Weighted, nonlinear least-squares fits of Equations S1 and S2 (see Text S9) derived from the binding models in parts (C) and (D) for R166S and S102G/R166S AP, respectively, are shown as solid lines. For R166S AP, 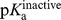 = 7.6±0.1 and 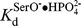 = 110±20 µM. For S102G/R166S AP, 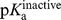 was fixed at 6.5 based on the tungstate binding data in part (B) and 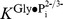
was fixed at 10^−6.1^ M based on the ^31^P NMR data in Figure 6. Fits yielded 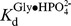 = 93±8 nM, and 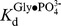 = 210±20 fM. (B) The pH-dependent binding of tungstate to R166S (open squares) and S102G/R166S AP (closed squares). A weighted, nonlinear least-squares fit of 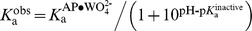 derived from a two-state tungstate binding model (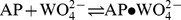) yielded a fit of the R166S AP data with 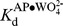 = 170±25 µM and 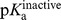 = 7.6±0.1. The corresponding fit to the observed tungstate affinity of S102G/R166S AP yielded a 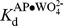 = 890±90 µM and 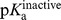 = 6.5±0.1. The tungstate affinity of S102G/R166S AP is weaker than R166S AP, indicating that Ser102 plays a favorable role in tungstate binding, possibly by allowing formation of octahedral tungstate as observed in other proteins that bind tungstate [73],[74]. At pH values ≥8 where the P_i_ affinity is strongest, the observed competition of ^32^P_i_ binding is likely influenced by competition from contaminating, unlabeled P_i_ in the tungstate stock rather than tungstate (see Materials and Methods). The dashed portion of the S102G/R166S AP tungstate fit line illustrates where the observed affinity can be accounted for by 0.5 ppm P_i_ contamination. Omitting the pH 9 and 10 points in the fit did not significantly change the fitted 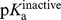 or 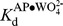 values. (C) Binding model used to fit the pH-dependent P_i_ affinity of R166S (and WT) AP. (D) Binding model used to fit the pH-dependent P_i_ affinity of S102G/R166S AP.

The pH dependence for the binding of P_i_ and other ligands can provide information about protonation events required for binding, which can in turn be used to distinguish between binding models. As previously reported for WT AP [20], and shown here for R166S AP, the pH dependence for binding of P_i_ follows a bell-shaped curve (Figure 4A, open symbols). At acidic pH, the observed P_i_ affinity decreases log-linearly with a slope of 1, indicating that a single protonation event greatly weakens or prevents binding. This protonation could prevent binding by either inactivating the enzyme or by forming a P_i_ species that does not bind. As the data are consistent with a p*K*_a_ of 6.9, which is the p*K*_a_ of 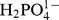 (at ionic strength, *I* = 0.1), these results suggest that R166S AP does not bind appreciably to 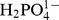.

A prediction of this model—that the p*K*_a_ of 6.9 is associated with the ligand and not the enzyme—is that this p*K*_a_ would be absent in the binding profile for a ligand that lacks a p*K*_a_ in this range. As predicted, the observed acidic limb with p*K*_a_ of 6.9 was not observed with tungstate (Figure 4B, open symbols), which has a p*K*_a_ below the pH range of our assays (p*K*_a_∼4 for 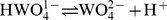 [40],[41]).

We next considered the basic limb of the bell-shaped pH-dependent profile for binding of P_i_ to R166S AP. The slope of −1 suggests that the loss of a single proton with a p*K*_a_ of 7.6 also prevents binding, and this result mirrors the behavior of WT AP [20]. Considering that P_i_ does not have a p*K*_a_ near 7.6 and that P_i_ and tungstate binding both have the basic limb with the same p*K*_a_ values of 7.6 (cf., R166S AP basic limbs in Figure 4A and B), this p*K*_a_ very likely represents an enzymatic deprotonation. One possible model for this binding deactivation involves deprotonation of a water molecule to leave a Zn^2+^-associated hydroxide ion blocking the bimetallo site of AP as suggested previously to account for the analogous pH-dependent loss of catalytic activity [20].

The above results are consistent with binding of 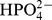 from solution. To determine whether there is additional binding from trianionic 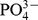 (
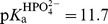
), binding assays were extended to higher pH values. Binding of 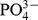 would manifest as an upturn in the basic limb of the pH dependence, but this limb remains linear with a slope of −1 in the highest pH region of 10–11.4 (Figure S9). These results provide no indication of AP binding 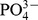 from solution and establish upper limits for the P_i_ dissociation constant 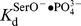 of 100 nM and 2.5 µM for WT AP and R166S AP, respectively (Table 2; Text S6; Figure S9).

**Table 2:** Summary of pH-independent P_i_ dissociation constants for AP active site nucleophile mutants.

Residue 102	Residue 166	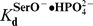 (M)^a^	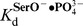 (M)^b^	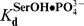 (M)^c^	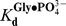 (M)^d^	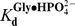 (M)^e^
Ser	Arg	4.6×10^−7^	≥1×10^−7^	≤2.9×10^−13^	—	—
Gly	Arg	—	—	—	*(∼1×10* ^−*15*^ *)*	*(∼1×10* ^−*8*^ *)*
Ser	Ser	1.1×10^−4^	≥2.5×10^−6^	≤6.9×10^−11^	—	—
Gly	Ser	—	—	—	2.1×10^−13^	9.3×10^−8^

**Figure 5:**
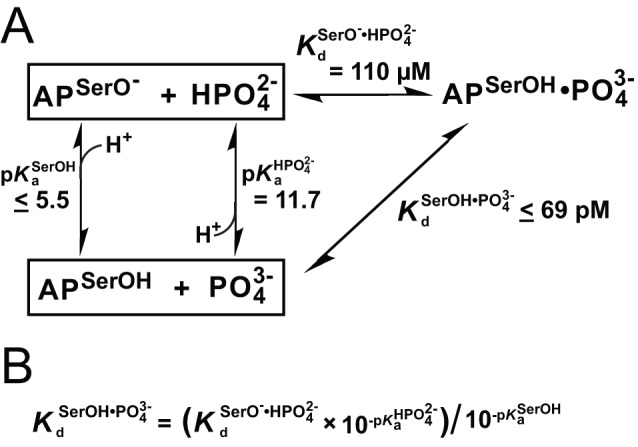
Thermodynamic cycle for 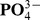 binding AP with Ser102 protonated. (A) The value of 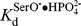 for R166S AP is from a fit of the model in Figure 4C to the pH-dependent P_i_ binding affinity in Figure 4A. The Ser102 p*K*_a_ is an upper limit [20], and thus, the dissociation constant between protonated Ser102 and 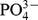 (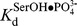) is also an upper limit (≤69 pM). The same cycle was used to establish an upper limit for the dissociation constant between WT AP with Ser102 protonated and 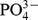, 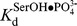
≤290 fM, from the following values: 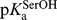
≤5.5, 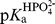 = 11.7, and 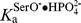 = 0.46 µM (Table 2). (B) Relationship derived from the thermodynamic cycle of part (A).

**—**, not applicable.

a Dissociation constant for 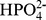 binding to deprotonated Ser102 as defined in Figure 4C from the measured pH-dependent P_i_ affinity in Figure 4A for R166S AP and Figure S9C for WT AP. Note that binding of 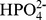 results in a proton transfer to the enzyme as illustrated in Equation 1; 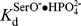 represents the observed overall binding.

b Lower limit of the dissociation constant for 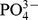 binding to deprotonated Ser102 AP determined as described in Text S6 and Figure S9.

c Upper limit for the dissociation constant for 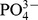 binding to protonated Ser102 AP as described in the main text and Figure 5.

d Dissociation constant for 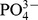 binding to S102G AP estimated from the measured 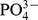 affinity of S102G/R166S AP and the expected contribution of Arg166 of 240-fold to the 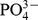 affinity (cf., 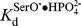
for WT and R166S AP). The dissociation constant for 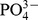 binding to S102G/R166S AP was determined from the pH-dependent binding data in Figure 4A and is defined in the model in Figure 4D.

e Dissociation constant for 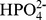 binding to S102G AP estimated based on an expected binding contribution from Arg166 of ∼10-fold (Text S12), and the dissociation constant for 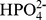 binding to S102G/R166S AP determined from the pH-dependent binding in Figure 4A; 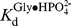 is defined in the model in Figure 4D.

The above results rule out significant binding of 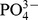 directly to AP with Ser102 intact. However, as noted above, our prior isotope-edited IR studies provide strong evidence for a proton transfer within the E•P_i_ complex to give bound 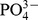 accompanied by protonation of a group on WT AP that was suggested to be Ser102 [16]. While analogous vibrational spectra could not be obtained for R166S AP due to signal-to-noise limitations, the observed ^31^P NMR chemical shift of P_i_ bound to WT and R166S AP is the same within error (3.8±0.2 and 3.7±0.2 ppm for R166S and WT AP, respectively; Text S7 and Figure S10), suggesting that the same P_i_ species is bound to both enzymes and allowing us to extend the assignment of bound 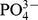 from WT AP to R166S AP.

The pH-dependent P_i_ and tungstate binding results, together with the previous vibrational spectroscopy measurements and ^31^P NMR comparison, lead to the model for P_i_ binding to R166S AP (and WT AP) shown in Figure 4C. A fit of this model to the pH-dependent P_i_ binding data for R166S AP shown in Figure 4A yields a pH-independent dissociation constant for 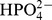 binding of 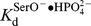 = 110 µM (Table 2) and a 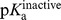 describing the basic limb of 7.6. This model further allowed us to assign intrinsic binding constants, as described below.

The thermodynamic cycle in Figure 5A can be used to compute a limit for the dissociation constant between 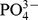 and the protonated form of Ser102, as binding of 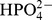 involves a formal internal proton transfer from 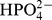 to Ser102 to give bound 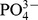. The relationship of Figure 5B, derived from Figure 5A, and the observed binding affinity of 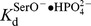 = 110 µM, the p*K*_a_ of 11.7 for 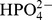, and the p*K*_a_ of ≤5.5 for deprotonation of Ser102 [20] give the affinity of R166S AP with protonated Ser102 for 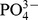, 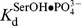
≤69 pM [ = ((110 µM)×(10^−11.7^ M))/(≥10^−5.5^ M); Table 2]. The calculation for WT AP was carried out analogously using the thermodynamic cycle in Figure 5A with the value of 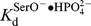 = 0.46 µM (Table 2) obtained by fitting the pH-dependent P_i_ affinity (Figure S9C; [16]) to the model in Figure 4C. Using this value and the relationship of Figure 5B yields the value for WT AP of 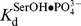
≤290 fM [ = ((0.46 µM)×(10^−11.7^ M))/(≥10^−5.5^ M)]; Table 2].

#### pH-dependent P_i_ binding to AP without the Ser102 nucleophile

We conducted pH-dependent P_i_ binding measurements with S102G/R166S AP using the ^32^P_i_ binding assay described above, as we could measure equilibrium binding constants with this AP mutant and not just limits as for S102G AP. The P_i_ affinity of S102G/R166S AP (Figure 4A) decreases log-linearly with a slope of 1 at acidic pH, indicating that a single protonation event prevents binding, as observed for R166S AP with Ser102 present. As 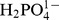 has a p*K*_a_ in the pH range of the observed acidic limb, it is likely that S102G/R166S AP does not have measurable affinity for 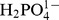, just like WT and R166S AP. Furthermore, as observed for R166S AP here and for WT AP previously [20], the pH dependence for S102G/R166S AP binding to tungstate, which does not have a p*K*_a_ in this range, lacks the acidic limb p*K*_a_ (Figure 4B), strongly supporting this interpretation.

The observed P_i_ binding affinity of S102G/R166S AP remains constant above pH 7 (Figure 4A). This dependence is different than that for WT and R166S AP, indicating that the protonation event(s) involved in P_i_ binding to S102G/R166S AP differ from those when Ser102 is present. The simplest model to account for the lack of an apparent basic limb for S102G/R166S AP would be that 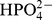 binds and the protein does not undergo the deprotonation that prevents binding as observed for WT and R166S AP. This model predicts that tungstate binding to S102G/R166S AP should also lack a basic limb. However, as shown in Figure 4B, tungstate binding decreases log linearly with a slope of 1 as the pH is raised, strongly suggesting that, like WT and R166S AP, S102G/R166S AP undergoes a pH-dependent loss of binding. The lower p*K*_a_ for S102G/R166S AP of 6.5, relative to 7.6 measured for R166S AP (Figure 4B), is consistent with more favorable formation of a Zn^2+^-associated hydroxide ion in the absence of the Ser102 anion. Nevertheless, our interpretations herein do not depend on the validity of this particular model for loss of binding at higher pH.

The absence of weaker binding of P_i_ as pH is increased indicates that the deactivating deprotonation of S102G/R166S AP revealed via tungstate binding is countered by a favorable deprotonation associated with P_i_ binding. This scenario is depicted in Figure 4D in which pH-independent binding arises from two counterbalancing deprotonation events, generation of inactive AP (at higher pH values), as noted above, and generation of the tighter binding species 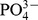 via deprotonation of the predominant 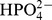 species at pH 7–10. Thus, S102G/R166S AP binds 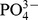 preferentially to 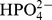, presumably by virtue of the increased negative charge of 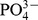 and the stronger net interactions with the positively charged AP active site. As elaborated below, this binding is much stronger than binding to R166S AP due to the absence of the negatively charged Ser102. These results also support our proposal that Ser102 acts as the proton acceptor in WT and R166S AP, because in its absence there is no internal proton transfer to the protein.

**Figure 6:**
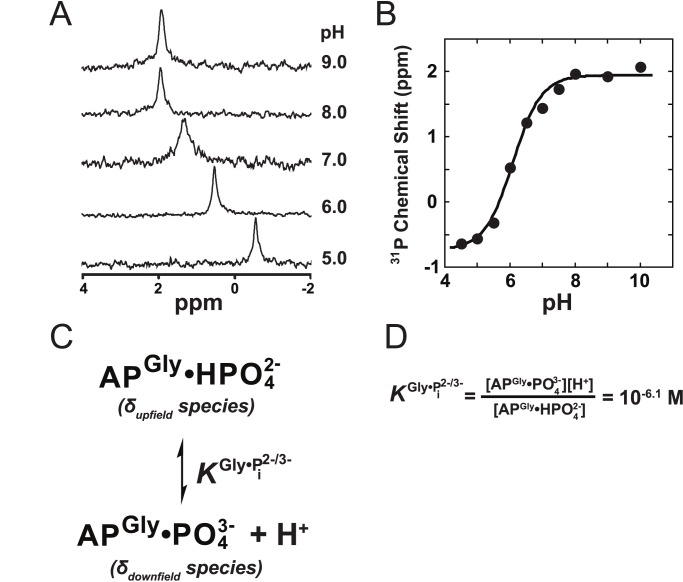
The pH-dependence of the ^31^P chemical shift of P_i_ bound to S102G/R166S AP. (A) ^31^P NMR spectra of P_i_ bound to S102G/R166S AP at various pH values. See Materials and Methods for conditions. (B) The chemical shift of P_i_-bound to S102G/R166S AP versus the solution pH. A nonlinear least-squares fit to an equation (see Materials and Methods) derived from the binding model in (C) yields δ_upfield_ and δ_downfield_ values of −0.74 and 1.94 ppm, respectively, and a value for 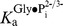 of 10^−6.1^ M as defined in (D).

While the above results indicate stronger binding of 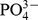 than 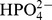, we considered whether S102G/R166S AP also has measurable binding of 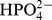. If 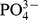 were the only species to detectably bind, we would expect to observe a single and constant ^31^P NMR chemical shift associated with S102G/R166S-bound 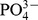 across all observable pH values. Instead, we observe that the chemical shift of P_i_ bound to S102G/R166S AP migrates as the solution pH is varied (Figure 6A), supporting a general model in which more than one P_i_ species binds with the bound species in fast exchange. Additional ^31^P NMR measurements with P_i_ in excess of AP demonstrated that the observed chemical shift variation does not reflect exchange between unbound and bound P_i_ (Text S8 and Figure S11). The observed chemical shift cannot be used to directly assign the ionic P_i_ form—the chemical shift does not correspond to that of any of the ionic forms in solution (Table S3), presumably due to active site interactions that are distinct from those in solution. Isotope-edited vibrational spectroscopy with P_i_ bound to S102G/R166S AP, as was carried out previously for WT AP [16], gave complex spectra (Figure S12) that could not be interpreted, presumably also due to the effects from the active site environment.

Although the bound P_i_ species could not be directly assigned via NMR or IR measurements, the pH dependence of binding provides strong evidence for the identity of the bound species. As noted above, the differential pH dependence of P_i_ and tungstate binding described above (Figure 4) strongly suggests that 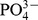 binds predominantly at pH values above 6.5. Further, the 2.7 ppm ^31^P chemical shift decrease for P_i_ bound to S102G/R166S AP upon going from high to low pH (Figure 6B) is similar to the decrease of 2.5 ppm upon protonation of 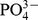 to give 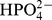 in aqueous solution [42]. Thus, the chemical shift at the lower pH likely arises from predominant binding of 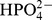. A fit to the pH dependence of the chemical shift migration yields a pH-dependent equilibrium constant (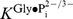) between S102G/R166S-bound 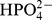 and 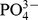 of 10^−6.1^ M (p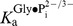 = 6.1; Figure 6B). This S102G/R166S AP-bound p*K*_a_ of 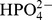 is 5.6 units lower than its solution p*K*_a_ of 11.7, reflecting the ability of the protein active site to strongly favor formation of 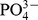 relative to solution.

P_i_ bound to S102G AP gives a constant ^31^P chemical shift from pH 4.5–10.2 with a value that is the same within error as the high-pH chemical shift of P_i_ bound to S102G/R166S AP (Figure S11A). This observation supports the above model and suggests, as expected, that Arg166 (S102G versus S102G/R166S AP) provides additional, preferential stabilization of bound 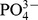 over 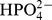.

Figure 4D shows the P_i_ binding model for S102G/R166S AP that incorporates all of the features noted above: an inactivating p*K*_a_, binding of 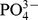 and 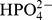, and protonation equilibria between both free and bound 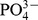 and 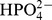. The equilibrium constants in this scheme were determined from the above-noted pH-dependent binding and NMR data (Figures 4 and 6), as described in the figure legend.

Our data also indicate that S102G AP binds P_i_ stronger than S102G/R166S AP, as expected from the additional interaction of Arg166 with the phosphoryl group (Figure 1). We could only obtain a lower limit for this affinity, but we could crudely estimate this value. Arg166 stabilizes 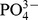 binding by 240-fold when Ser102 is present [(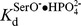)^R166S^/(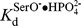)^WT^ = 110 µM/0.46 µM = 240; Table 2] and a similar stabilization would be expected when Ser102 is absent because the same interactions can form. We thus estimate a dissociation constant of ∼1 fM [(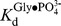)^S102G/R166S^/240 = 210 fM/240 = 0.9 fM] for 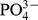 binding to S102G AP (Table 2). While this is a crude estimate, the affinity is clearly in the range of the strongest measured in biology [17],[43]. In addition, while known ligands with femtomolar binding affinities contain at least 16 atoms [43], 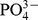 has just five atoms, albeit with high charge density, and only four directly interacting with the AP active site.

**Figure 7:**
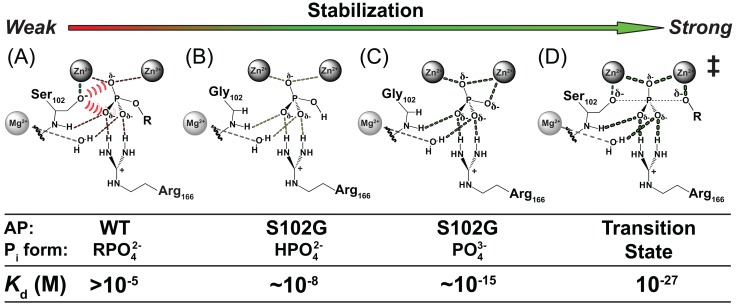
Models for AP binding P_i_ dianion and trianion and summary of AP binding energetics. (A) The dissociation constant limit for dianionic phosphate monoester binding to WT AP was determined in ref. [20]. This limit is also expected to hold for R166S AP where an interacting residue is removed. (B) Removing Ser102 strengthens binding of a dianion by ≥10^3^-fold, as 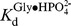 was estimated to be ∼10 nM (Table 2). (C) Trianion binding (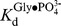; Table 2) was estimated to be ∼1 fM and is 10^7^-fold stronger than dianion binding. (D) The AP rate enhancement is 10^27^-fold (for methyl phosphate dianion hydrolysis: *k*_cat_/*K*_M_ = 1.2×10^6^ M^−1^ s^−1^ [20] and *k*_uncat_∼4×10^−22^ M^−1^ s^−1^ [75]), corresponding to a theoretical dissociation constant for transition state binding of 10^−12^ fM (derived in Figure S1 of ref. [16]). This theoretical affinity reflects binding of the enzyme to the transition state while accompanied by replacement of water by the active site Ser102 nucleophile. The energetics of these two processes cannot be separated and the formal dissociation constant reflects contributions from both binding interactions and positioning of the Ser102 nucleophile and substrate.

The enormous rate enhancement that AP provides by interacting with the transferred phosphoryl group transition state suggests that this enzyme is capable of making very strong interactions. Indeed, the ∼1 fM binding of 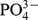 shown here illustrates that a substantial portion of the transition state stabilization energy can be unmasked and manifested in the ground state by removing the Ser102 alkoxide. The substantial additional affinity for the transition state presumably arises from positioning of the reacting Ser102 and phosphoryl group as well as optimized positioning of interacting groups to better complement the trigonal bipyramidal geometry of the transition state versus the tetrahedral geometry of the 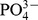 ground state (cf., models for the 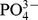 ground state and the transition state in Figure 7).

### Destabilization of 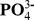 Binding by the Ser102 Nucleophile

With the binding affinities determined for individual P_i_ species (Table 2), we were able to determine a minimum amount for the destabilization of 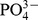 binding caused by the Ser102 alkoxide. We compared the 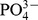 affinity in AP lacking Ser102 to AP with the deprotonated Ser102 alkoxide intact. In the absence of the Ser102 alkoxide, the 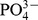 dissociation constant is 210 fM and ∼1 fM for S102G/R166S and S102G AP, respectively. Limits for the 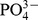 affinity of AP with deprotonated Ser102 (+/− Arg166) were estimated from the absence of detectable P_i_ binding at high pH (Figure S9), as described above, and give lower limits for the 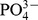 dissociation constant (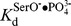) of ≥2.5 µM and ≥100 nM for R166S and WT AP, respectively, and thus, upper limits for the affinity. Comparing these affinity limits to the 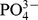 affinity in the absence of Ser102 reveals a destabilization from the Ser102 alkoxide of at least 10^7^–10^8^ fold (for S102G/R166S AP, *K*_rel_ = ≥2.5 µM/210 fM; for S102G AP, *K*_rel_ = ≥100 nM/∼1 fM). (For a further comparison of the 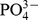 affinity in the presence of protonated Ser102 and with Ser102 mutated to Gly, see Text S10 and Figure S13 therein.)

### Destabilization of Dianion Substrate Binding by the Ser102 Nucleophile

Our ability to measure the 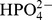 affinity of S102G/R166S AP, with a dissociation constant of 90 nM, allows us to estimate the Ser102 destabilization to a dianionic phosphate monoester substrate, as 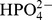 and a phosphate ester have the same overall charge and tetrahedral geometry. (See Text S11 for discussion of a previous [16] estimation of the Ser102 destabilization of dianion binding.) In comparison to WT AP with Ser102 intact, which has a dissociation constant for dianionic substrate binding of >10 µM [20], S102G/R166S AP binds a dianion at least 10^2^-fold more strongly (>10 µM versus 90 nM), suggesting a destabilization of substrate (E•S) binding from the Ser102 alkoxide of at least 10^2^-fold. Analogous considerations lead to a suggested destabilization of at least 10^3^-fold with Arg166 present (WT versus S102G AP) as the 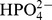 affinity of S102G is expected to be ∼10-fold stronger than the 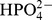 affinity of S102G/R166S AP (see Text S12). From a practical perspective, if Ser102 did not provide this destabilization, the enzyme would saturate with substrate concentrations of ∼10 nM, at least 10^3^-fold lower than the *K*_M_ with destabilization from Ser102 present. Such a low *K*_M_ would greatly limit turnover and function any time substrate concentrations exceeded the *K*_M_.

The 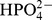 affinity in the absence of Ser102 is very strong, but the 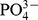 affinity is much stronger—at least 10^7^ fold stronger (Table 2; 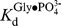/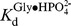 for S102G AP). In Text S13 we discuss potential origins of this enhanced affinity.

In addition to destabilizing substrate binding, Ser102 also destabilizes the binding of the reaction product, P_i_, by ∼10^3^-fold at pH 8.0 (Table 1), thereby preventing subnanomolar product inhibition. This consideration, along with the analysis of substrate destabilization above, strongly suggests that ground state destabilization from an anionic nucleophile can make a substantial catalytic contribution and suggests that other phosphatases with anionic nucleophiles may also exhibit ground state electrostatic repulsion.

### Active Site Nucleophile Destabilization in a Protein Tyrosine Phosphatase

To test whether the results in AP generalize to other phosphatases with negatively charged active site nucleophiles, we compared the P_i_ affinity of a protein tyrosine phosphatase (PTP) with and without its active site nucleophile. The PTP Stp1 is a member of the low-molecular weight PTP family, which uses a negatively charged cysteine nucleophile [44]. With its Cys11 nucleophile intact, we measured a dissociation constant for P_i_ binding by Stp1 of 18 mM at pH 6.0 (Table 3; Figure S14), in reasonable agreement with a previous measurement [45]. Measurements were carried out at pH 6.0 because this is the pH of maximal catalytic activity [46]. When Cys11 is mutated to Gly, P_i_ binding gets stronger, with a dissociation constant of 10 µM, demonstrating a 10^3^-fold destabilizing influence from the Cys11 nucleophile. The observed destabilization effect of Ser102 in AP (R166S) at pH 6 is also ∼10^3^-fold (cf., data points at pH 6 in Figure 4A). In the absence of extensive pH-dependent binding studies, we cannot assign the ionic form of P_i_ that binds Stp1 as was done for AP and mutants thereof. Nevertheless, our findings of analogous increases in affinity upon removal of Cys11 from Stp1 supports a model in which this anionic active site nucleophile destabilizes ground state binding, and raises the possibility that ground state destabilization is a general strategy among phosphatases with anionic nucleophiles.

**Table 3:** Binding of P_i_ to a PTP with and without its active site Cys anionic nucleophile.

Protein	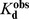 (µM)^a^	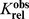 b
Wild type	(1.8±0.4)×10^4^	(1)
C11G	10±5	1,800

a See Materials and Methods for assay conditions.

b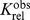 is the ratio of the observed P_i_ dissociation constant in the presence of Cys11 compared to the dissociation constant in the absence of Cys11: *K*_d_(Cys11)/*K*_d_ (Gly11).

### Preferential Ground State Destabilization from Ser102

We propose that the destabilization from electrostatic repulsion by Ser102 is present in the substrate (E•S) and product (E•P_i_) ground states and is absent, or nearly so, in the reaction's transition state. Recall that in order for ground state destabilization to play a role in catalysis, the destabilization must not be present in the transition state (as illustrated in Figure 2D). Specific destabilization of the ground state results in a lowering of the transition state barrier and catalysis of the chemical step is thereby accelerated.

Previous studies of phosphoryl transfer reactions in solution [47]–[49] provide strong evidence against substantial electrostatic repulsion in the transition state, suggesting that electrostatic repulsion in the enzymatic ground state does not carry over into the transition state as required for ground state destabilization to be catalytic. Text S14 provides a summary of these studies.

### Summary and Implications

To accelerate chemical reactions, enzymes must provide stabilization to the reaction's transition state yet limit binding to ground states—i.e., substrates and products. As noted in the Introduction, AP imparts an exceptional rate enhancement to the hydrolysis of phosphate monoesters, corresponding to a formal stabilization of the transition state of 10^27^-fold. The same active site that provides this enormous transition state stabilization limits ground state binding to an affinity of at most 10 µM, which is more than 10^22^-fold weaker than the formal transition state stabilization (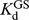
>10^−5^ M versus 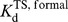 = 10^−27^ M; Figure 1).

The similarity of the transition and ground states of the AP-catalyzed reaction (Figure 1) raises the question of how AP distinguishes so profoundly between these states and, in particular, how AP specifically limits ground state binding. Our results support a model in which electrostatic repulsion from the anionic active site Ser102 nucleophile plays an important role in limiting ground state binding.

The most common source of preferential ground state destabilization in enzyme active sites, as described in the Introduction, is presumably the ubiquitous entropic cost incurred upon binding free substrates and positioning them with respect to catalytic residues in the enzyme active site (and to each other for multisubstrate reactions). Other sources of ground state destabilization have also been suggested when binding energy, in addition to paying for the entropic penalty of binding, is used to impart geometrical distortion (typically referred to as “strain”) or electrostatic destabilization to the bound ground state. Approaches including X-ray crystallography (e.g., [50]–[52]), vibrational spectroscopy (for review, see [53]), and binding isotope effect measurements (e.g., [54]–[59]) have identified enzyme-bound substrates (or analogs thereof) in alternative, or distorted, conformations relative to the corresponding structures in solution. These distortions in the ground state tend to approach the conformation thought to be present in the transition state, leading to the proposal that such distortions contribute to catalysis. While our understanding of transition state structures and properties are well enough advanced that many of these proposals are likely correct, they do not reveal the underlying energetics of the destabilizing distortion or the specific residues responsible for imparting the distortion (see also [60]).

We have combined binding, structural, and spectroscopic studies of AP to obtain a quantitative energetic estimate of ground state destabilization and have assigned this effect to a particular active site residue, the active site nucleophile Ser102. Similar destabilization was shown for an unrelated phosphatase, PTP, and is likely present in the many other classes of phosphoryl transfer enzymes that use anionic nucleophiles or metal-coordinated anionic hydroxide.

The ≥10^3^-fold electrostatic ground state destabilization from anionic nucleophiles ascribed herein is one component of the overall transition state stabilization conferred by AP and other phosphatases. As illustrated in Figure 7, even after removing the ≥10^3^-fold destabilization from Ser102, binding is still much weaker compared to the formal transition state stabilization implied by the 10^27^-fold rate enhancement that AP provides relative to the corresponding reaction in water. Thus, destabilization from Ser102 is just one component that, together with other active site features and properties of AP, imparts the overall observed catalysis, as is consistent with the general view that enzymes catalyze reactions through multiple mechanisms and interactions, each with a relatively modest contribution [4],[23]. Text S15 presents further discussion of these other catalytic mechanisms and provides additional context for the observations herein.

## Materials and Methods

### AP Expression and Purification

Mutant and WT AP were purified using an N-terminal maltose binding protein (MBP) fusion construct (AP-MBP) in the pMAL-p2X vector (New England Biolabs), as previously described [37]. Purity was estimated to be >95% as judged visually by band intensities on Coomassie blue-stained SDS-polyacrylamide gels. Protein concentrations were determined by absorbance at 280 nm (background subtracted by absorbance at 330 nm) in 8 M guanidine hydrochloride (Gdn•HCl) using a calculated extinction coefficient for the AP monomer of 31,390 M^−1^ cm^−1^ [61]. Concentrations of active WT AP and R166S AP were confirmed by activity assays using 1 mM *p*-nitrophenyl phosphate (*p*NPP) and agreed with previously reported *k*_cat_ values [35] to within 20%.

Following purification, the ratio of AP to P_i_ present was approximately 0.6 for WT AP and 0.95–1 for the Ser102 AP mutants. The fractional P_i_ content was reduced to below 0.1 by dialysis in 6 M Gdn•HCl at 25°C for several days, as previously described [16]. R166S AP did not have associated P_i_ after purification but was still subjected to the same dialysis procedure as the other AP variants. For WT and R166S AP, activity assays using *p*NPP demonstrated that at least 90% of the pre-dialyzed activity was retained. For the Ser102 mutants, which lacked measurable activity, the post-dialyzed samples were capable of stoichiometric binding of P_i_, similarly indicating that there was no significant loss in P_i_ binding activity from the dialysis procedure.

### P_i_ Binding Affinity Measurements

AP•P_i_ affinities were previously determined using kinetic inhibition assays, typically by inhibition of *p*NPP activity or promiscuous *p*-nitrophenyl sulfate (*p*NPS) activity (e.g., [16],[20],[62]). The observed low level of activity of the Ser102 mutant preparations is likely due to contaminating phosphatase activity (Text S1), and thus, inhibition of this activity would not reflect binding to the Ser102 mutants. Consequently, a new equilibrium-binding assay was developed that enabled the determination of P_i_ dissociation constants. For this assay, ^32^P_i_ (∼200 pM or less; Perkin Elmer NEX053002MC) was added to samples containing varying concentrations of AP (with less than 0.1 fraction pre-bound P_i_) in the standard buffer conditions of 100 mM buffer, 100 mM NaCl, 1 mM MgCl_2_, and 100 µM ZnCl_2_ at 4°C. The following buffers were used over the indicated pH ranges: NaAcetate (4.5–5.5), NaMES (5.5), NaMaleate (6.0–6.5), Tris•HCl (7.0–7.5), NaMOPS (7.0–8.0), NaCHES (8.5–9.0), and NaCAPS (10–10.5).

For equilibrium measurements, after a period sufficient to allow equilibration as demonstrated by achieving constant binding over time, each AP sample was subjected to brief filtration through a 10 kDa molecular weight cutoff centrifugal filter (VWR centrifugal filters, modified PES, 10K, 500 µL) by centrifugation at 3,600 *g* for ∼90 s. (Binding of P_i_ to S102G and S102A AP likely does not reach equilibration within the time course of the assay; limits for their P_i_ affinities were estimated using the kinetics of P_i_ uptake and dissociation as described in Text S3 and figures therein.) The filtrate volume (10–50 µL), which was much smaller than the retentate volume (400 µL) so as to avoid significant changes in the protein concentration in the retentate, is expected to contain free ^32^P_i_. The retentate is expected to contain both free and bound ^32^P_i_. No significant AP (<0.1%) passed through the filter as assessed by activity assays of the filtrate of WT and R166S AP samples. Variation of the filtration time and volume did not result in significant differences in the concentration of ^32^P_i_ passing through the filter, suggesting that bound ^32^P_i_ is not significantly lost over the time of centrifugation. Scintillation counting of both the filtrate and retentate was used to measure the concentrations of free and bound ^32^P_i_ at the various AP concentrations, and the fraction ^32^P_i_ bound dependence on the AP concentration was used to determine dissociation constants using a modified binding equation, *f*_bound_ = (*C*)×([AP]/([AP]+*K* 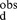))+(1–*C*), where *C* allows for background levels of apparent binding in the absence of protein. Samples containing no protein usually gave fraction ^32^P_i_ values very close to 0, although background levels ranging from −0.02 to 0.15 were observed in some instances (see Figures S1A, S8A, S8C, and S8E).

An alternative assay was used in some instances to lower background levels of apparent P_i_ binding and thereby provide greater sensitivity to small amounts of bound P_i_. Filtration units that contained G-25 Sephadex (USA Scientific) in the top portion to trap unbound ^32^P_i_ and allow bound ^32^P_i_ to pass through always trapped ∼100% of the unbound ^32^P_i_ so that when no protein is present the background fraction binding observed is very close to 0. However, very high protein concentrations did not reach 100% binding as expected, but only approached 85%–90% bound—presumably from loss of ^32^P_i_ bound to AP as the bound complex passes through the Sephadex resin during the filtration. The uptake and dissociation of ^32^P_i_ measured with these filters (*vide infra*) give results in agreement with the uptake and dissociation of ^32^P_i_ results obtained using the membrane filtration method described above (see Figure S8I–L).

To measure the uptake of ^32^P_i_ by the AP sample, the fraction of ^32^P_i_ bound was measured over time starting just after the addition of ^32^P_i_ to the AP samples. The uptake was fit to the single exponential equation 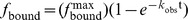. Plotting *k*_obs_ versus the AP concentration allowed *k*_on_ and *k*_off_ values to be determined by fitting the data to the equation for bimolecular binding rates of 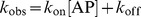 (see Table S1 for kinetic values determined for each AP mutant). For S102G and S102A AP, the amount of ^32^P_i_ uptake observed was much less than expected, potentially reflecting a protein inactivation process. Fitting of a model allowing for irreversible protein inactivation to the P_i_ binding kinetics for these mutants was conducted using the KinTek Global Explorer program [63],[64] as described in the Text S3 and Figure S4 (see also Figures S5 and S6).

Although *k*_off_ values can in principle be determined from the uptake assays, these values, determined by the y-intercept of plots of the observed uptake rate constant versus the concentration of protein, are highly sensitive to small errors in the slope (*k*_on_). We used chase assays to independently determine *k*_off_ for P_i_ binding, which were conducted by first incubating ^32^P_i_ with concentrations of AP sufficient to result in near complete ^32^P_i_ binding, and then after incubation times long enough to allow equilibration, saturating levels of unlabeled P_i_ well above the AP and ^32^P_i_ concentrations were added. The concentration of the unlabeled P_i_ addition was varied (2–20 mM) to ensure saturation and the absence of any secondary effects. Immediately following the addition of unlabeled P_i_, the filtration procedure was used to determine the fraction ^32^P_i_ bound. As ^32^P_i_ dissociates from the protein it is replaced by unlabeled P_i_ and the observed fraction of ^32^P_i_ decreases. The time-dependent loss of the fraction ^32^P_i_ bound was fit to a single exponential decay equation 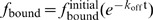.

The WT Stp1•P_i_ affinity was determined using a kinetic inhibition assay. The *p*NPP hydrolysis activity of Stp1 was measured in 20 mM NaMaleate, 100 µM Na_2_EDTA, and 0.15 M NaCl at pH 6.0 and 4°C in the absence and presence of P_i_ inhibitor. A range of P_i_ concentrations was added to the kinetic assays from at least 5-fold below to 5-fold above the inhibition constant. The concentration of Stp1 was 20 nM and the concentration of *p*NPP was 50 µM (5-fold below the *K*_M_ under these conditions so that the *K*_i_ essentially equals the *K*_d_ for P_i_ binding). Nonlinear least-squares fits of the equation for competitive inhibition [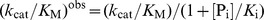] gave fits with standard errors of less than 10% (Figure S14B).

The C11G Stp1•P_i_ affinity was determined using the equilibrium-binding assay that was used for AP described above. The buffer conditions, pH, and temperature were the same as those for the WT Stp1 kinetic inhibition assays (Figure S14A).

### Tungstate Affinity Measurements

The binding of tungstate to the S102G/R166S AP mutant was measured using a variation of the equilibrium-binding assay described above in which observed ^32^P_i_ binding is competed with tungstate. Various concentrations of tungstate (at least 5-fold above and below the expected binding dissociation constant) were first incubated with a concentration of S102G/R166S AP needed to achieve ∼0.5 fraction ^32^P_i_ binding with no tungstate present under the standard buffer conditions at 4°C. Variation of the incubation time of S102G/R166S AP with tungstate from 1–6 h did not affect the observed competition binding. After this first incubation, trace ^32^P_i_ was added and the sample was incubated further to allow ^32^P_i_ binding to complete. The resulting dependence of the tungstate concentration on the observed fraction ^32^P_i_ bound was well fit to the simple binding isotherm, 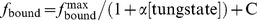, where α is the *K*_a_ of tungstate binding in the limit that the free tungstate concentration is equal to the total tungstate concentration and C is the background fractional ^32^P_i_ binding as the tungstate concentration approaches infinity (<0.15). The tungstate competition assays had 0.5–10 µM S102G/R166S AP, the concentration needed to achieve ∼0.5 fraction ^32^P_i_ binding depending on the pH; at most, 20% of the total tungstate concentration is bound over all conditions so that [tungstate]_free_≈[tungstate]_total_. This competition assay reproduced well the pH-dependent tungstate affinity of WT AP that was previously measured using kinetic inhibition methods (Figure S15; [20]).

As noted in the legend of Figure 4B, the observed tungstate affinity of S102G/R166S AP at pH values ≥8 deviates from the log-linear affinity decrease expected for a protein inactivation with p*K*_a_∼6.5. The observed competition measured at these pH values likely originates from contaminating levels of unlabeled P_i_ in the tungstate stock. The deviation is consistent with the constant P_i_ affinity in this pH range (Figure 4A) with P_i_ present in only one part in ∼10^6^ (i.e., the observed tungstate affinity of S102G/R166S AP in Figure 4B at pH values ≥8 is approximately 10^6^-fold lower than the P_i_ affinity over the same pH values in Figure 4A). Assays of the tungstate stock for P_i_, using malachite green [65], resulted in a very high absorbance signal, likely because tungstate itself can form a complex with malachite green and mask the signal from relatively very low levels of P_i_ contamination.

The tungstate affinity for R166S AP was determined by inhibition of *p*NPP hydrolysis as was done previously with WT AP [20], but under the standard buffer conditions used in this work. A range of tungstate concentrations was used from at least 5-fold below and above the inhibition constant at each pH.

### Crystallization, Structure Determination, and Refinement

S102G/R166S AP (23.5 mg/mL in 10 mM NaMOPS, pH 8.0, and 50 mM NaCl) was crystallized at 18°C by the sitting-drop method using a mother liquor of 0.2 M NH_4_F, 17%–21% PEG (polyethylene glycol) 3350, and 500 µM ZnCl_2_ (conditions adapted from [37]). Crystals were passed through a 30% glycerol solution in mother liquor before direct immersion in liquid nitrogen.

Diffraction data were collected at the Stanford Synchrotron Radiation Lightsource on beamline 11-1. Data were integrated and scaled using DENZO and SCALEPACK, respectively [66], and 5% of data were set aside for cross-validation [67]. Data statistics are summarized in Table S2.

Initial phases were determined by molecular replacement with Phaser [68] using R166S AP (PDB entry 3CMR; [35]) as a search model, with Ser102 truncated to a glycine. Subsequently, σ_A_-weighted 2*F*_o_–*F*_c_ and *F*_o_–*F*_c_ maps were inspected, and a complete model comprising residues 4–449 and three Zn^2+^ ions per monomer. Model building was performed using Coot [69].

In most structures of AP, two Zn^2+^ ions occupy the bimetallo site and a Mg^2+^ ion occupies the third metal site. The high ZnCl_2_ concentrations used in the crystallization conditions here apparently allow Zn^2+^ to outcompete Mg^2+^ for the third metal site. Mg^2+^ was not included in the crystallization conditions. An alignment with a previously determined AP structure with Mg^2+^ bound in the third metal site showed no significant structural differences of the coordinating ligands at the metal site regardless of whether Zn^2+^ or Mg^2+^ is occupying the site, suggesting that Zn^2+^ replacement of Mg^2+^ has very limited structural consequences on residues beyond this metal ion coordination sphere (see Figure S16 for structural overlay). This metal ion is 4.6 Å from the closest oxygen atom of the bound P_i_ in WT AP (3TGO; [36]).

Noncovalently bound phosphate was modeled in the active site of S102G/R166S AP to account for the appearance of tetrahedral electron density there, as in prior structures 1ALK [33] and 3TGO [36]. Although no P_i_ was added during the crystallization, P_i_ copurifies with S102G/R166S AP, binds the protein tightly, and contaminates commercial PEG solutions (Hampton Research) used for the crystallization [37].

From this model, maximum-likelihood amplitude-based refinement was carried out using refmac [70], resulting in an *R*-factor of 23.0% and *R*_free_ of 31.0%. Final stages of refinement were carried out with Force Field X [71]. Each stage of refinement was interspersed with manual corrections and model adjustments using Coot. The *R* and *R*_free_ values for the final refined model were 23.2% and 29.6%, respectively. All structural figures were prepared using MacPyMol [72].

### ^31^P NMR

Samples for ^31^P NMR measurements had 1–2 mM AP, 100 mM buffer, 100 mM NaCl, 1 mM MgCl_2_, and 100 µM ZnCl_2_. Sub-stoichiometric and excess levels of P_i_ were added to the samples to identify peaks that were associated with bound-P_i_ and free-P_i_ in solution. ^31^P NMR spectra were recorded at 161.97 MHz on a Varian Mercury spectrometer equipped with a broadband tunable probe. Protein samples of ∼350 µL were contained in 5 mm tubes fitted with a coaxial capillary insert (Wilmad Lab Glass) containing D_2_O for the external field-frequency lock. Spectra were recorded at 37°C with a sweep width of 50,000 Hz, pulse delay of 2 s, and an acquisition time of 0.8 s. Proton decoupling was employed and S/N of >10 could usually be obtained after ∼10,000 transients (∼11 h). A line broadening of 5–10 Hz was typically applied and all spectra were referenced to a 1% phosphoric acid external standard.

The observed ^31^P chemical shift data for S102G/R166S AP-bound P_i_ as a function of pH (Figure 6) was fit to δ_obs_ = (δ_upfield_−δ_downfield_)/(1+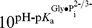)+δ_downfield_, which was derived from a two-state model in which an upfield and downfield species bind in a ratio dependent on the solution pH (Figure 6C). The 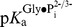 value is the p*K*_a_ of the equilibrium between 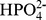-bound S102G/R166S AP and 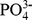-bound S102G/R166S AP (as defined in Figure 6C and D).

### Accession Numbers

Protein Data Bank Code for the S102G/R166S AP X-ray crystal structure: 4KM4.

## Supporting Information

Figure S1Tests of the new equilibrium-binding assay with WT AP. (A) Three replicate equilibrium-binding assays for ^32^P_i_ binding at pH 8.0 (in standard buffer conditions, see Materials and Methods) gave nonlinear least-squares fits for fractional ^32^P_i_ binding with 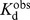 = 0.20±0.02 µM (diamonds, dashed line), 0.24±0.05 µM (circles, solid line), and 0.34±0.06 µM (triangles, dotted line). The average *K*_d_ value from the three assays is 0.26±0.07 µM (Table 1 and Table S1). Samples of WT AP and ^32^P_i_ were incubated for >1 h before the fraction ^32^P_i_ bound was measured and no dependence on the incubation time was observed (not shown). (B) Inhibition of *p*NPP hydrolysis activity by P_i_ under the standard reaction conditions at pH 8.0 with 0.4 µM *p*NPP. Activity was normalized by dividing the observed rate constant in the presence of inhibitor by the rate constant in the absence of inhibitor. Two replicate assays yielded individual, nonlinear least-squares fits for competitive inhibition with *K*_d_ values of 0.67±0.04 µM (circles) and 0.67±0.06 µM (diamonds); the combined fit is shown with *K*_d_ = 0.67±0.04 µM. (C) Inhibition of *p*NPS hydrolysis activity by P_i_ under the standard reaction conditions at pH 8.0 with 21 mM *p*NPS. To achieve activity significantly above background, a much higher concentration of AP (0.2 µM) is needed than for the *p*NPP inhibition assays. As a result, the simplifying assumption that [inhibitor]_free_ = [inhibitor]_total_ does not hold; [AP] is similar to the expected *K*_i_ and the commonly used form of the Michaelis–Menten equation with competitive inhibition could not be used. Instead, a quadratic equation (below) was used to relate the observed fractional activity to the total inhibitor and AP concentrations used in the assay and a nonlinear least-squares fit of this equation gave *K*_d_ = 0.50±0.14 µM. (D) The equilibrium-binding assay at pH 8.0, 9.5, and 10.5 with 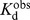 values of 0.24 µM (circles), 1.7 µM (diamonds), and 23 µM (triangles), respectively. (E) Comparison of the pH-dependent 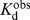 values from the new equilibrium-binding assay [closed circles; part (D)] with values measured by kinetic inhibition (open circles; from Figure S9C). The line shown is the combined fit of both data sets and gives a protein inactivation p*K*_a_ value of 8.7, in agreement with the inactivation p*K*_a_ value determined over the full pH range (Figure S9C). (F) The fraction ^32^P_i_ bound determined by the filtration assay after addition of unlabeled P_i_ (2 mM) to AP•^32^P_i_ (assay in standard buffer conditions with 10 µM WT AP), giving a limit for *k*_off_ of ≥0.01 s^−1^. (G) The uptake of ^32^P_i_ after addition of ^32^P_i_ to P_i_-free AP and following the fraction bound using the filtration assay in standard buffer conditions with 0.1–10 µM WT AP; k
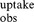
≥0.03 s^−1^ for all concentrations of WT AP.
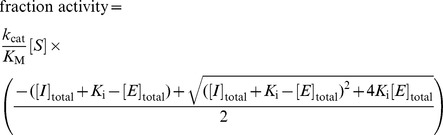

(EPS)

Figure S2Equilibrium-binding assay with S102G and S102A AP. All assays were carried out at pH 8.0 under the standard buffer conditions described in Materials and Methods. (A) Dissociation of ^32^P_i_ from S102G and S102A AP. Excess unlabeled P_i_ (2 mM) was added to 1 µM samples of S102G (open diamonds) or S102A (closed circles) AP with maximal ^32^P_i_ bound. After the addition of unlabeled P_i_, the fraction ^32^P_i_ bound was measured over time. The line shows the expected behavior for a dissociation rate constant of 2×10^−7^ s^−1^ (t_1/2_∼1,000 h), which provides the upper limit used in the text. (B, C) Uptake of ^32^P_i_ over time by S102G (B) or S102A (C) AP. (P_i_-free proteins were generated as described in Materials and Methods.) Nonlinear least-squares fits to the equation 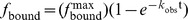 at each protein concentration were used to estimate the observed uptake rate constant and the endpoint ^32^P_i_ fraction bound. The observed background (at 0 min) fraction ^32^P_i_ bound is different for the two uptake assays because different filtration devices were used for each assay (see Materials and Methods). (D, E) The observed uptake rate constants versus each S102G (D) or S102A (E) AP concentration. For simple two-state binding, *k*_obs_ = *k*_on_[AP]+*k*_off_ [23]. The weighted, nonlinear least-squares fits shown as solid lines give slope values (*k*_on_) of 1,300 and 1.6×10^4^ M^−1^ s^−1^ for S102G and S102A AP, respectively. The *y*-intercept values for these plots are strongly influenced by small errors in the slope fit and are not typically interpreted for this reason. Fixing the *y*-intercept value at the limit for *k*_off_ suggested by the chase assays (2×10^−7^ s^−1^) nevertheless yields reasonable fits to the data as shown by the dashed lines. Note the log scale in (D) but not in (E).(EPS)

Figure S3 Dilution of S102G AP after uptake of ^32^P_i_. S102G AP (1 µM at pH 8.0 in standard buffer conditions) was incubated with ∼200 pM ^32^P_i_ for 400 min. The sample was serially diluted 2-fold by addition of buffer at *t* = 400, 1,430, and 1,810 min (as indicated by the vertical dashed lines) to give the S102G AP concentrations indicated at the top of the figure, and the fraction ^32^P_i_ bound was measured after each dilution using the filtration assay.
(EPS)

Figure S4 Model and fits for uptake of ^32^P_i_ by S102G and S102A AP. (A) The model for uptake of ^32^P_i_ with both reversible binding of ^32^P_i_ and irreversible inactivation of the free protein. *k*_off_ was fixed at the limit measured with the chase assay [the fits in parts (B) and (C) were insensitive to lowering *k*_off_ below the upper limit of 2×10^−7^ s^−1^]. (B) The uptake of ^32^P_i_ by S102G AP (data from Figure S2B) fit to the model in part (A). For fitting, the data were normalized to correct for the ∼0.1 fraction ^32^P_i_ loss during centrifugation for the filtration units used in these assays (see Materials and Methods). The lines show a global, nonlinear fit for *k*_on_ and *k*_inactive_ and yielded values of ∼1,000 M^−1^ s^−1^ and ∼3×10^−4^ s^−1^, respectively. (C) The uptake of ^32^P_i_ by S102A AP (data from Figure S2C) fit to the model in part (A). For model fitting, the data were normalized to correct for the background fraction binding at time zero of ∼0.1, as observed for the type of filtration units used in this assay (see Materials and Methods). The global fit shown to the model in (A) yielded a poor fit, particularly to the data at lower concentrations of S102A AP.
(EPS)

Figure S5 Initial uptake rate constant analysis for S102G and S102A AP. (A) The uptake of ^32^P_i_ by S102G AP (data from Figure S2B). The dashed lines are the single exponential fits to the entire uptake time course, as shown in Figure S2B. The solid lines estimate the rate of initial ^32^P_i_ uptake. (B) The estimated initial uptake rate constants from the slopes of the lines in (A) at each concentration of S102G AP. The nonweighted, linear fit shown gives a slope of *k*_on_ 740 M^−1^ s^−1^ (with the *y*-intercept, *k*_off_, fixed at 2×10^−7^ s^−1^). Note the logarithmic *x*-axis scale. (C) The uptake of ^32^P_i_ by S102A AP (data from Figure S2C). The dashed lines are the single exponential fits to the entire uptake time course, as shown in Figure S2C. The solid lines estimate the rate of initial ^32^P_i_ uptake. (D) The estimated initial uptake rate constants from the slopes of the lines in (C) at each concentration of S102A AP. The nonweighted, linear fit shown gives a slope for *k*_on_ of 1.2×10^4^ M^−1^ s^−1^ (with the *y*-intercept, *k*_off_, fixed at 2×10^−7^ s^−1^).
(EPS)

Figure S6 Equilibrium-binding assays for S102G and S102A AP. Samples of various concentrations of S102G (A) or S102A (B) AP were incubated with ∼200 pM ^32^P_i_ at pH 8.0 in the standard buffer conditions. The samples were incubated ≥24 h before the fraction ^32^P_i_ bound was measured. Independent replicate assays are depicted by different symbols. Fits to a simple two-state binding isotherm yielded a variable midpoint of the fraction bound values and yielded a steeper binding dependence than expected for simple 1∶1 binding. As noted in Text S3, these and other results suggest complications for protein inactivation over the long times of these assays and led us to use weaker binding and faster equilibrating mutants for quantitative comparisons.
(EPS)

Figure S7 Inhibition by P_i_ and equilibrium-binding assay for R166S AP. (A) Inhibition of R166S AP *p*NPP hydrolysis activity by P_i_ at pH 8.0 under the standard reaction conditions (see Materials and Methods) with [*p*NPP] = 0.8 µM. Three independent replicate assays are shown. Activity was normalized by the observed rate constant in the absence of inhibitor. The lines are nonlinear least-squares fits for competitive inhibition and give an average *K*_i_ value of 360±160 µM. (B) Equilibrium-binding assay conducted for R166S AP at pH 8.0 (closed circles) and 9.0 (open diamonds) under the standard reaction conditions. The dashed line shows the predicted fraction ^32^P_i_ bound for a dissociation constant of 360 µM.
(EPS)

Figure S8P_i_ equilibrium-binding assay for S102G/R166S and S102A/R166S AP. (A) Three independent equilibrium-binding assays for ^32^P_i_ binding to S102G/R166S AP at pH 8.0 (in standard buffer conditions). Nonlinear least-squares fits for fractional ^32^P_i_ binding give an average 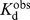 value of 66±8 nM. Incubation times were >300 min. (B) The fraction ^32^P_i_ bound followed by the filtration assay after addition of unlabeled P_i_ (2 mM) to 1 µM S102G/R166S AP pre-bound with ^32^P_i_ (assay in standard buffer conditions). The line shows a nonlinear least-squares fit with a first-order decay constant (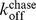) of 1.2±0.05×10^−4^ s^−1^. (C) Uptake of ^32^P_i_ over time by S102G/R166S AP followed by the filtration assay. Nonlinear least-squares fits at each protein concentration were used to estimate the observed uptake rate constant and the endpoint ^32^P_i_ fraction bound. (D) The observed uptake rate constants versus S102G/R166S AP concentration. The weighted, nonlinear least-squares fit shown as a solid line yields a *k*_on_ value of 1,190±120 M^−1^ s^−1^. Fixing the *y*-intercept value at the *k*_off_ determined in the chase assay (1.2×10^−4^ s^−1^) yields the fit shown by the dashed line and does not significantly change the *k*_on_ value obtained. The largest deviations from this fit occur at the lowest concentrations of S102G/R166S AP, which have the highest *k*_obs_ fit error as illustrated by the error bars (assuming symmetrical error). (E) Three independent equilibrium-binding assays for ^32^P_i_ binding to S102A/R166S AP at pH 8.0. Nonlinear least-squares fits for fractional ^32^P_i_ binding give an average 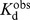 value of 77±6 nM. Incubation times were >4 d. (F) The fraction ^32^P_i_ bound followed by the filtration assay after addition of unlabeled P_i_ (2 mM) to 1 µM S102A/R166S AP pre-bound with ^32^P_i_ (assay in standard buffer conditions). The line shows a nonlinear least-squares fit with a first-order decay constant (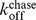) of 1.6±0.06×10^−6^ s^−1^, assuming a background fraction-bound at *t* = ∞ of 0.05, as was observed for the S102G/R166S AP chase assay. (G) Uptake of ^32^P_i_ over time by S102A/R166S AP followed by the filtration assay. Nonlinear least-squares fits at each protein concentration were used to estimate the observed uptake rate constant and the endpoint ^32^P_i_ fraction bound. (H) The observed uptake rate constants versus S102A/R166S AP concentration. The weighted, nonlinear least-squares fit shown as a solid line gives a *k*_on_ value of 51±4 M^−1^ s^−1^. Fixing the *y*-intercept value at the *k*_off_ determined in the chase assay (1.6×10^−6^ s^−1^) yields the fit shown by the dashed line and does not significantly change the *k*_on_ value obtained. The largest deviations of this fit occur at the lowest concentrations of S102A/R166S AP, which have the highest *k*_obs_ fit error as illustrated by the error bars. (I) Uptake of ^32^P_i_ over time by S102G/R166S AP followed using a filtration device containing G-25 Sephadex resin (see Materials and Methods). These filtration devices give very low background ^32^P_i_ fraction binding (but note that the maximum ^32^P_i_ bound that could be observed only approaches 0.85–0.90, presumably from loss of ^32^P_i_ bound to AP as the bound complex passes through the Sephadex resin during the filtration). Nonlinear least-squares fits at each protein concentration were used to estimate the observed uptake rate constant and the endpoint ^32^P_i_ fraction bound. (J) The observed uptake rate constants versus S102G/R166S AP concentration using filtration devices containing G-25 Sephadex resin. The weighted, nonlinear least-squares fit shown as a solid line gives a *k*_on_ value of 1,420±520 M^−1^ s^−1^, within error of the value obtained with the filtration method used in part (C). Fixing the *y*-intercept value at the *k*_off_ determined in the chase assay (1.6×10^−6^ s^−1^) yields the fit shown by the dashed line and does not significantly change the *k*_on_ value obtained. The largest deviations of this fit occur at the lowest concentrations of S102G/R166S AP, which have the highest *k*_obs_ fit error as illustrated by the error bars. (K) Uptake of ^32^P_i_ over time by S102A/R166S AP followed using filtration devices containing G-25 Sephadex resin. Nonlinear least-squares fits at each protein concentration were used to estimate the observed uptake rate constant and the endpoint ^32^P_i_ fraction bound. (L) The observed uptake rate constants versus S102A/R166S AP concentration using filtration devices containing G-25 Sephadex resin. The weighted, nonlinear least-squares fit shown as a solid line gives a *k*_on_ value of 36±14 M^−1^ s^−1^ within error of the value obtained with the filtration devices used in part (G). Fixing the *y*-intercept value at the *k*_off_ determined in the chase assay (1.6×10^−6^ s^−1^) yields the fit shown by the dashed line and does not significantly change the *k*_on_ value obtained. The largest deviations of this fit occur at the lowest concentrations of S102A/R166S AP, which have the highest *k*_obs_ fit error as illustrated by the error bars. The fit error for the 0.2 µM S102A/R166S AP uptake sample is exceptionally large, reflecting the influence of an outlier data point collected during the time course.(EPS)

Figure S9pH-dependent P_i_ binding affinity to WT AP and R166S AP to estimate 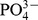 affinity to AP with Ser102 deprotonated. Assays in standard conditions of 100 mM NaAcetate (pH 4.5–5.5), NaMaleate (pH 6.0–6.5), NaMOPS (pH 7–8.0), NaCHES (pH 8.5–9.5), NaCAPS (pH 10–11.2), or NaCABS (pH 11.0–11.4), with 100 mM NaCl, 1 mM MgCl_2_, and 100 µM ZnCl_2_. (A) The model used to estimate the 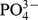 binding affinity. (B) The *p*NPP hydrolysis activity of R166S AP from pH 7–10. The line shows the expected trend for a single enzymatic inactivation given by 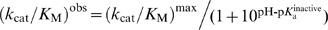 with 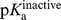 = 7.6 and (*k*_cat_/*K*_M_)^max^ = 8×10^4^ M^−1^ s^−1^. (C) The pH-dependent P_i_ affinity of WT AP. Weighted, nonlinear least-squares fits of Equation S3 with various fixed values of *K* 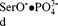 are shown. 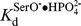 was fixed at 0.46 µM and 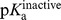 was fixed at 8.6, which are the values from the optimal fits assuming no 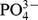 affinity. The fit using a fixed *K* 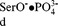
of 100 nM deviated 4-fold from the data above pH 11.0. (D) The pH-dependent P_i_ affinity of R166S AP. Weighted, nonlinear least-squares fits of Equation S3 with various fixed *K* 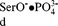 values are shown. *K* 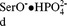 was fixed at 110 µM and 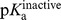 was fixed at 7.6, which are the values from the optimal fits assuming no 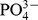 affinity. The fit with a *K* 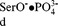 = 2.5 µM deviates from the data at pH 10 with greater than the expected error of these measurements (<50%).
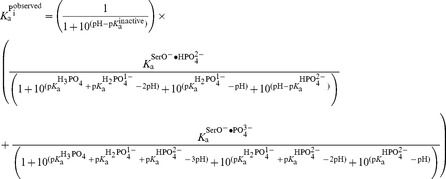
(Equation S3)
(EPS)

Figure S10 ^31^P NMR of P_i_ with R166S AP. (A) Samples contained ∼1.3 mM R166S AP, 10 mM NaTris, pH 7.5, 100 mM NaCl, 1 mM MgCl_2_, and 100 µM ZnCl_2_. The concentration of P_i_ was initially very low to achieve the highest fraction bound. The concentration of P_i_ was subsequently increased by adding small volumes of concentrated P_i_ to achieve the lower fraction bound values listed. The fraction P_i_ bound was computed using the measured R166S AP dissociation constant for P_i_ binding of 360 µM. ^31^P NMR parameters are as described in Materials and Methods. The chemical shift approaches the shift of the bound P_i_ shift observed in WT AP (bottom spectrum). (B) Extrapolation of the ^31^P NMR chemical shift as the fraction P_i_ bound increases yields a chemical shift for bound P_i_ of ∼3.8 ppm.
(EPS)

Figure S11 ^31^P NMR spectra of S102G (A) and S102G/R166S (B) AP with excess P_i_. Solution conditions for all ^31^P NMR measurements are described in Materials and Methods. Protein concentrations were typically 1–2 mM and P_i_ concentrations were approximately 2-fold in excess. When P_i_ is not in excess, only the chemical shift peaks demarcated with red arrows remain present (spectra not shown). The additional peaks present with P_i_ in excess correspond to the chemical shifts expected for the unbound species of P_i_ at each pH (see Table S3).
(EPS)

Figure S12 [^18^O]-P_i_ edited FTIR difference spectra of P_i_ in solution and P_i_ bound to WT, S102G, and S102G/R166S AP. (A) FTIR difference spectrum between ^16^O-P_i_ and ^18^O-P_i_ at pH 4.5 (green), 9.5 (blue), or 13 (red). (B) FTIR difference spectra between AP•^16^O-P_i_ and AP•^18^O-P_i_ for WT AP (2.6 mM AP/2.3 mM P_i_) in 140 mM NaMOPS, pH 8.0, 680 mM NaCl, 1.4 mM MgCl_2_, and 140 µM ZnCl_2_ (from ref. [16]); S102G AP (3.4 mM AP/1 mM P_i_) in 110 mM NaCHES, pH 9.5, 100 mM NaCl, 1 mM MgCl_2_, and 100 µM ZnCl_2_; and S102G/R166S AP (2.2 mM AP/1 mM P_i_) in 50 mM NaCHES, pH 9.0, 100 mM NaCl, 1 mM MgCl_2_, and 100 µM ZnCl_2_. All samples and spectra were prepared and acquired as described in [16].
(EPS)

Figure S13Comparison of 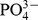 binding of AP with and without Ser102. Comparison of 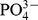 binding AP with Ser102 either mutated to Gly, deprotonated or protonated. Black bars show the 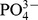 affinity measured for S102G/R166S AP and estimated for S102G AP (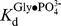 values in Table 2 of the main text). Red bars show the upper limits for the 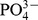 affinity (denoted by downward arrows) measured for R166S and WT AP with Ser102 deprotonated (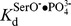 values in Table 2 of the main text). Grey bars show the lower limits for the 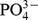 affinity for R166S and WT AP with Ser102 protonated (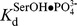 values in Table 2 of the main text).(EPS)

Figure S14 Binding of P_i_ to a protein tyrosine phosphatase with and without its nucleophilic cysteine present. (A) Equilibrium-binding assay of the PTP, Stp1, with Cys11 intact (open circles) and three independent assays of C11G Stp1 (closed symbols) at pH 6.0 in 20 mM NaMaleate, 100 µM Na_2_EDTA, and 0.15 M NaCl at 4°C. Fits to the data for C11G Stp1 gave *K*_d_ values of 4.9±0.5 µM (inverted triangles), 9.6±1.7 µM (squares), and 15±2.3 µM (triangles). The average *K*_d_ value from the three assays is 10±5 µM. The observed fraction ^32^P_i_ bound did not depend on incubation times of C11G Stp1 with ^32^P_i_ greater than 60 min. No significant P_i_ binding of WT Stp1 could be detected within the protein concentration limits of the assay (500 µM). [In contrast to the observations with R166S AP at pH 8.0, concentrations of Stp1 >25 µM did not result in a significant decrease in flow rate through the filtration unit (see Text S4).] (B) Inhibition of WT Stp1 *p*NPP hydrolysis activity by P_i_ at pH 6.0 in 20 mM NaMaleate, 100 µM Na_2_EDTA, and 0.15 M NaCl for three independent assays. Fits yield *K*_i_ values of 14±4 mM (circles), 19±2 mM (open triangles), and 20±2 mM (open diamonds) with an average *K*_i_ value of 18±3 mM. WT and C11G Stp1 were purified as reported previously [44].
(EPS)

Figure S15The pH dependence of tungstate binding to WT AP. (A) Fraction ^32^P_i_ bound to AP as a function of tungstate concentration. A competition-binding assay at pH 8.0 for WT AP (see Materials and Methods for assay details) under the standard buffer conditions. A nonlinear least-squares fit to the competition equation shown in Materials and Methods yields a dissociation constant value for tungstate binding (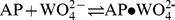) of 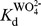 = 0.9±0.2 µM in good agreement with the dissociation constant at this pH reported previously (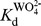 = 1.1 µM; [20]) at pH 8.0. (B) The pH-dependent tungstate affinity for WT AP determined by kinetic inhibition measurements (circles) and by the equilibrium-binding assay via competition with ^32^P_i_ binding (squares). The fits shown yield inactivating p*K*_a_ values of 7.82±0.13 (solid line) and 8.07±0.16 (dashed line) and pH-independent dissociation constants for tungstate dianion binding of 0.57±0.11 µM (solid line; data from [20]) and 2.06±0.48 µM (dashed line).(EPS)

Figure S16 Structural overlay of AP Mg^2+^ site. The WT AP crystal structure (PDB code 3TGO, [36]) is shown in grey with the crystal structure reported here for S102G/R166S AP in magenta. In the S102G/R166S AP structure, a Zn^2+^ ion occupies the Mg^2+^ site. The position of the coordinating residues and the metal ion are the same within error in the two structures.
(EPS)

Table S1Summary of P_i_ binding kinetics for WT, S102G, S102A, R166S, S102G/R166S, and S102A/R166S AP. ^a^ *k*_on_ for S102G and S102A AP is the estimated association rate constant from a fit analysis of the ^32^P_i_ uptake assay results described in Text S2. *k*_on_ for S102G/R166S and S102A/R166S AP is from the fit of the *k*_obs_ values from the uptake assay versus the [AP] shown in Figure S8D and H. ^b^ 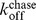 is the dissociation rate constant measured by the ^32^P_i_ chase assay (Figure S1F; Figure S2A; Figure S8B and F). ^c^ 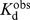 is the dissociation constant for P_i_ binding at pH 8.0 measured from the fraction ^32^P_i_ bound after an incubation time sufficient to reach equilibrium (Figure S1A for WT AP; Figure S8A for S102G/R166S; Figure S8D for S102A/R166S AP), except for the value reported for R166S AP, which is from kinetic inhibition assays (Figure S7A). ^d^The 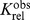 value is calculated by dividing the dissociation constant for AP with Ser102 intact by the dissociation constant (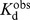) for the Ser102 mutants in either the context of WT or R166S AP; larger values represent stronger binding of the Ser102 mutant relative to proteins with Ser102 intact.(DOC)

Table S2 Crystallographic data and model statistics.
(DOC)

Table S3 ^31^P NMR chemical shift summary of free P_i_ and P_i_ bound to WT, R166S, S102G, and S102G/R166S AP. ^a^Chemical shifts reported for unbound P_i_ species were measured here under conditions identical to those used for protein-containing samples and referenced to a 1% phosphoric acid standard. These shifts are within error of those reported previously [42]. At intermediate pH values the observed chemical shift represents a weighted average of the ionic forms present.
(DOC)

Text S1 Observed activity of Ser102 mutants likely arises from WT AP contamination.
(DOC)

Text S2 Tests of the new equilibrium-binding assay with WT AP.
(DOC)

Text S3 Equilibrium-binding assay results with S102G and S102A AP.
(DOC)

Text S4 Equilibrium binding of P_i_ to R166S, S102G/R166S, and S102A/R166S AP.
(DOC)

Text S5 Interplay between Ser102 and Arg166 revealed by structural comparison.
(DOC)

Text S6Estimation of 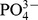 affinity for AP with Ser102 deprotonated.(DOC)

Text S7
^31^P NMR of the R166S AP•P_i_ complex suggests bound 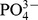.(DOC)

Text S8 ^31^P NMR of P_i_-bound to S102G and S102G/R166S AP with excess P_i_.
(DOC)

Text S9 Equations derived from the models in Figure 4C and D to fit the pH-dependent P_i_ binding data for R166S (Equation S1) and S102G/R166S (Equation S2) AP in Figure 4A.
(DOC)

Text S10 Comparison of AP•P_i_ affinities with AP Ser102 protonated, deprotonated, or mutated to Gly.
(DOC)

Text S11 Previous estimation for the destabilization from Ser102 on the binding of a dianionic phosphate.
(DOC)

Text S12 Estimation of the contribution of Arg166 to binding of P_i_ dianion.
(DOC)

Text S13Comparison of 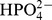 and 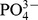 binding by S102G AP.(DOC)

Text S14 Evidence against electrostatic repulsion in phosphoryl transfer transition states.
(DOC)

Text S15 Extended implications for phosphoryl transfer catalysis.
(DOC)

## Abbreviations

AP: alkaline phosphatase
P_i_: inorganic phosphate
PTP: protein tyrosine phosphatase
