# Supplementary material for: Ground State Destabilization by Anionic Nucleophiles Contributes to the Activity of Phosphoryl Transfer Enzymes
Source: PLoS Biol. 2013 Jul 2;11(7):e1001599. doi: 10.1371/journal.pbio.1001599 (PMC3699461; doi:10.1371/journal.pbio.1001599)
Supplement: Table S1 — Summary of Pi binding kinetics for WT, S102G, S102A, R166S, S102G/R166S, and S102A/R166S AP. a k on for S102G and S102A AP is the estimated association rate constant from a fit analysis of the 32Pi uptake assay results described in Text S2. k on for S102G/R166S and S102A/R166S AP is from the fit of the k obs values from the uptake assay versus the [AP] shown in Figure S8D and H. b is the dissociation rate constant measured by the 32Pi chase assay (Figure S1F; Figure S2A; Figure S8B and F). c is the dissociation constant for Pi binding at pH 8.0 measured from the fraction 32Pi bound after an incubation time sufficient to reach equilibrium (Figure S1A for WT AP; Figure S8A for S102G/R166S; Figure S8D for S102A/R166S AP), except for the value reported for R166S AP, which is from kinetic inhibition assays (Figure S7A). dThe value is calculated by dividing the dissociation constant for AP with Ser102 intact by the dissociation constant () for the Ser102 mutants in either the context of WT or R166S AP; larger values represent stronger binding of the Ser102 mutant relative to proteins with Ser102 intact. (DOC) [file pbio.1001599.s017.doc]

**Table S1.** Summary of P_i_ binding kinetics for WT, S102G, S102A, R166S, S102G/R166S, and S102A/R166S AP

| **AP** | ***k*_on_**^(a)^ **(M^-1^s^-1^)** | ***k***^(b)^ **(s^-1^)** | ***k*/*k*_on_ (nM)** | ***K***^(c)^ **(nM)** | ***K***^(d)^ |
| --- | --- | --- | --- | --- | --- |
| WT | - | ≥0.1 | - | 260 (±74) | (1) |
| S102G | ~1000 | ≤2×10^-7^ | ≲0.2 | - | ≳1000 |
| S102A | ~1×10^4^ | ≤2×10^-7^ | ≲0.02 | - | ≳1×10^4^ |
| R166S | - | - | - | (3.6 ± 1.6) ×10^5^ | (1) |
| S102G/R166S | 1190 (±120) | (1.2 ± 0.05) × 10^-4^ | 103 (±15) | 66 (±8) | 5500 |
| S102A/R166S | 51 (±4) | (1.6 ± 0.06) × 10^-6^ | 32 (±4) | 77 (±6) | 4700 |

^a^ *k*_on_ for S102G and S102A AP is the estimated association rate constant from a fit analysis of the ^32^P_i_ uptake assay results described in Text S2. *k*_on_ for S102G/R166S and S102A/R166S AP is from the fit of the *k*_obs_ values from the uptake assay versus the [AP] shown in Figure S8D and H. ^b^ is the dissociation rate constant measured by the ^32^P_i_ chase assay (Figure S1F; Figure S2A; Figure S8B and F). ^c^$K_{d}^{\text{obs}}$ is the dissociation constant for P_i_ binding at pH 8.0 measured from the fraction ^32^P_i_ bound after an incubation time sufficient to reach equilibrium (Figure S1A for WT AP; Figure S8A for S102G/R166S; Figure S8D for S102A/R166S AP), except for the value reported for R166S AP, which is from kinetic inhibition assays (Figure S7A). ^d^ The value is calculated by dividing the dissociation constant for AP with Ser102 intact by the dissociation constant ($K_{d}^{\text{obs}}$) for the Ser102 mutants in either the context of WT or R166S AP; larger values represent stronger binding of the Ser102 mutant relative to proteins with Ser102 intact.
