# Supplementary material for: Ground State Destabilization by Anionic Nucleophiles Contributes to the Activity of Phosphoryl Transfer Enzymes
Source: PLoS Biol. 2013 Jul 2;11(7):e1001599. doi: 10.1371/journal.pbio.1001599 (PMC3699461; doi:10.1371/journal.pbio.1001599)
Supplement: Table S2 — Crystallographic data and model statistics. (DOC) [file pbio.1001599.s018.doc]

**Table S2.** Crystallographic data and model statistics

|  | S102G/R166S AP |
| --- | --- |
| *Data collection* |  |
| Beamline | SSRL 11-1 |
| Wavelength (Å) | 0.9795 |
| Space group | *P*6_3_22 |
| Unit cell dimensions (Å) |  |
| *a* | 160.935 |
| *b* | 160.935 |
| *c* | 139.549 |
| Resolution range (Å) | 50-2.8 |
| Effective resolution (Å) (*I / σ* =2) | 50-2.9 |
| No. of total reflections | 4,616,524 |
| No. of unique reflections | 26,488 |
| Completeness (highest-resolution shell) | 99.2 (98.5) |
| Redundancy (highest-resolution shell) | 20.4 (19.4) |
| *I / σ* (highest-resolution shell) | 7.3 (1.5) |
| *R*_merge_ (%)^a^ | 33.7 |
|  |  |
| *Refinement statistics* |  |
| *R*-factor (*R*_free_) (%)^b^ | 23.2 (29.6) |
| No. of protein atoms | 6546 |
| No. of solvent atoms | 0 |
| No. of ligand atoms | 16 |
| Average *B*-factor | 21.5 |
| RMSD bond lengths (Å) | 0.013 |
| RMSD bond angles (°) | 2.6 |
| ^a^ *R*_merge_ = | |
| ^b^ *R*-factor = See the work of Brunger for a description of *R*_free_; ref. [25] | |
