# Supplementary material for: Ground State Destabilization by Anionic Nucleophiles Contributes to the Activity of Phosphoryl Transfer Enzymes
Source: PLoS Biol. 2013 Jul 2;11(7):e1001599. doi: 10.1371/journal.pbio.1001599 (PMC3699461; doi:10.1371/journal.pbio.1001599)
Supplement: Table S3 — 31P NMR chemical shift summary of free Pi and Pi bound to WT, R166S, S102G, and S102G/R166S AP. aChemical shifts reported for unbound Pi species were measured here under conditions identical to those used for protein-containing samples and referenced to a 1% phosphoric acid standard. These shifts are within error of those reported previously [42]. At intermediate pH values the observed chemical shift represents a weighted average of the ionic forms present. (DOC) [file pbio.1001599.s019.doc]

**Table S3.** ^31^P NMR chemical shift summary of free P_i_ and P_i_ bound to WT, R166S, S102G, and S102G/R166S AP

| **Protein-Bound P_i_ AP Species** | **pH** | **^31^P NMR chemical shift (ppm)** |
| --- | --- | --- |
| WT | 5.0-9.0 | 3.7 |
| R166S | 7.5 | 3.8 |
| S102G | 4.5-10.2 | 1.94 |
| S102G/R166S | >8.0 | 1.94 |
| S102G/R166S | <5.0 | -0.74 |
| **Free P_i_ Species**^(a)^ |  |  |
| H_2_PO | 4.0 | 0.05 |
| HPO | 7.5 | 2.19 |
| PO | 13.0 | 4.64 |

^a^ Chemical shifts reported for unbound P_i_ species were measured here under conditions identical to those used for protein-containing samples and referenced to a 1% phosphoric acid standard. These shifts are within error of those reported previously [28]. At intermediate pH values the observed chemical shift represents a weighted average of the ionic forms present.
