# Supplementary material for: Ground State Destabilization by Anionic Nucleophiles Contributes to the Activity of Phosphoryl Transfer Enzymes
Source: PLoS Biol. 2013 Jul 2;11(7):e1001599. doi: 10.1371/journal.pbio.1001599 (PMC3699461; doi:10.1371/journal.pbio.1001599)
Supplement: Text S1 — Observed activity of Ser102 mutants likely arises from WT AP contamination. (DOC) [file pbio.1001599.s020.doc]

**Text S1. Observed activity of Ser102 mutants likely arises from WT AP contamination**

AP mutants with the Ser102 nucleophile removed have been reported to retain a low level of phosphate monoester hydrolysis activity [1,2]. However, our results provide strong evidence that this activity arises from contaminants in the mutant preparations.

The reported activity of the Ser102 mutant preparations from different laboratories vary by 40-fold under similar conditions. Values for *k*_cat_/*K*_M_ for the hydrolysis of *p*-nitrophenyl phosphate (*p*NPP) by S102A AP of 30 M^-1^s^-1^ [1], 1.3×10^3^ M^-1^s^-1^ [2], and 10-1000 M^-1^s^-1^ from preparations in our lab have been observed. Observation of these kinetic parameters would require WT AP to be present at only 0.01-0.0001% [(*k*_cat_/*K*_M_)^WT AP^ ~ 1×10^7^ M^-1^s^-1^; e.g., [3-6]]. The prior studies of S102A AP gave inhibition constants for P_i_ that were similar to the inhibition constant obtained for WT AP in these studies (*K*_i_ = 15 and 30 μM in [1] measured for WT and S102A AP, respectively; *K*_i_ = 5.6 ± 0.7 μM and 7.9 ± 0.3 μM in [2] measured for WT and S102A AP, respectively), as would be expected if a small fraction of WT AP were responsible for the observed activity. These reported *K*_i_ values are greater than the actual dissociation constant for P_i_ binding to WT AP likely because the *p*NPP substrate was present at concentrations greater than its *K*_M_ and because inhibiting concentrations of P_i_ would have been generated in the course of the reaction (see ref. [6]).

The results of our current study provide additional, strong evidence that the Ser102 mutant enzymes are not responsible for the observed activity. Our P_i_ binding results indicate that the Ser102 mutants would be strongly inhibited even with sub-micromolar levels of P_i_ (*K*_i_ values at pH 8 range from ≲0.02 – 80 nM for the Ser102 mutants used herein; see Table 1 and Table S1). For example, the amount of P_i_ giving half-inhibition in the prior studies (~10 μM) would give a least 10^5^-fold inhibition (*K*_i_ ≲ 0.02 nM with S102A AP; Table S1), not the 2-fold inhibition that was observed. Further, spectroscopic activity assays of phosphate monoesters [6] require substrate concentrations that would produce highly inhibitory concentrations of P_i_ and thus result in minimal turnover and strong curvature if the activity arose from the mutant AP; e.g., full turnover of 200 nM of *p*NPP, resulting in the production of 200 nM P_i_, gives a change of only 0.003 AU (=16,652 M^-1^cm^-1^ for *p*-nitrophenolate at pH 8.0), which is near or below typical spectrophotometer noise levels. Thus, even if the mutant APs have some residual activity, negligible product accumulation would be observed from their activity, and thus, the reported and observed activities must arise from contaminating activities.
