# Supplementary material for: Ground State Destabilization by Anionic Nucleophiles Contributes to the Activity of Phosphoryl Transfer Enzymes
Source: PLoS Biol. 2013 Jul 2;11(7):e1001599. doi: 10.1371/journal.pbio.1001599 (PMC3699461; doi:10.1371/journal.pbio.1001599)
Supplement: Text S2 — Tests of the new equilibrium-binding assay with WT AP. (DOC) [file pbio.1001599.s021.doc]

**Text S2. Tests of the new equilibrium-binding assay with WT AP**

The affinity of ground state ligands for AP is typically determined by measuring inhibition of catalytic activity (with *p*NPP, or sometimes *p*-nitrophenyl sulfate, *p*NPS) under conditions in which the observed *K*_i_ equals the *K*_d_ for ligand binding (e.g., [6-9]). To obtain accurate measurements of P_i_ binding, the overall turnover of the substrate, which results in the generation of P_i_, should be less than the *K*_i_ value of P_i_. When more P_i_ is generated during the course of the reaction, the enzyme is subject to additional product inhibition and the observed inhibited activity is a complex combination of the amount of P_i_ added and the amount generated during the assay. For WT AP, the *p*NPP substrate concentration used during inhibition measurements (~0.5 μM) is kept near, but below the observed *K*_i_ for P_i_ binding (0.5-1 μM) at pH 8.0, thereby avoiding substantial product inhibition even when the reaction is allowed to go to completion while enabling a change in absorbance that can be accurately followed (full turnover of 0.5 μM of substrate results in a 0.008 AU change; $e_{400}^{\text{max}}$=16,652 M^-1^cm^-1^ for *p*-nitrophenolate at pH 8.0).

The observed binding of P_i_ to the Ser102 AP mutants at pH 8.0 is much stronger than to WT AP. Consequently, to accurately measure the P_i_ affinity of these mutants, much less substrate would need to be used. Unfortunately, as noted above, the limits of detecting product require substrate concentrations of at least 0.2-0.5 μM. As shown herein, the inhibition constant for P_i_ at pH 8.0 is ≲0.2 nM for the S102G and S102A AP mutants and ~75 nM for the S102G/R166S and S102A/R166S AP mutants, preventing measurement via inhibition of activity. As also noted above (Text S1), there is no evidence of any measurable activity from the Ser102 mutants of AP and the activity that is observed likely arises from trace WT AP contamination.

To measure P_i_ binding to the Ser102 mutants a new equilibrium-binding assay was developed. The details of this assay are described in the Methods section of the main text. Briefly, the assay entails incubating various concentrations of AP with trace ^32^P_i_. The bound and unbound populations of AP are then separated using a centrifugal filter, and the fraction ^32^P_i_ bound is measured using scintillation counting of the filtrate and the retentate.

To test the validity of this assay, several controls were carried out. First, the fraction of ^32^P_i_ bound to WT AP was plotted as a function of the WT AP concentration and the data fit to a simple binding isotherm. Figure S1A shows three replicate binding assays. The dissociation constant obtained was ~2-fold lower than that from previous measurements using inhibition of activity, agreement that is reasonable given the errors in these assays and the difficulty of obtaining accurate measurements in the kinetic assay due to the low substrate concentrations needed (*K*_d_ = 0.26 ± 0.074 μM from the new equilibrium-binding assay, Figure S1A; *K*_d_ = 0.61 ± 0.1 μM from kinetic inhibition assays of *p*NPP and *p*NPS, Figure S1B and C).

To further test the new equilibrium-binding assay, the P_i_ affinity of WT AP was measured at higher pH. The binding of P_i_ by WT AP was previously shown to be pH-dependent [6,7] (see also Figure S9C): as the pH is raised from ~8, the observed P_i_ affinity decreases log linearly with a slope of -1. The results using the new assay for P_i_ binding to WT AP at pH 8.0, 9.5, and 10.5 are shown in Figure S1D, and the *K*_d_ values are plotted in Figure S1E along with the previously determined values measured by kinetic inhibition [7]. The results agree well, demonstrating that the new assay is capable of measuring the AP•P_i_ affinities as weak as ~10 µM.

As an additional test of the new equilibrium-binding assay, the kinetics of P_i_ release and uptake were measured using this assay. To measure P_i_ release, ^32^P_i_ was first incubated with a concentration of WT AP high enough to achieve near-complete ^32^P_i_ binding. After this incubation, an excess of unlabeled P_i_ (≥2 mM) was added and the fraction ^32^P_i_ bound was assessed at different times after this unlabeled P_i_ chase. As shown in Figure S1F, all of the ^32^P_i_ was released before the first time point after the addition of unlabeled P_i_, giving a lower limit of the release rate constant, *k*_off_, of ≥0.01 s^-1^, consistent with an estimate of *k*_off_ ~ 10 s^-1^ from previous studies [10-12]. Analogous results were obtained for the kinetics of ^32^P_i_ uptake (Figure S1G), which is also expected to be fast relative to the time resolution of the assay. (The observed uptake for a reversible binding process is the sum of *k*_on_[AP] + *k*_off_ [13]: for 0.1 μM AP, *k*_obs_ = (0.1 μM)*k*_on_ + 10 s^-1^ and the expected *k*_on_ value is ~1×10^7^ M^-1^s^-1^ [5,6,14]. Thus, the expected *k*_obs_ at this protein concentration is 11 s^-1^ –a value that is faster than the time resolution of the assay used here and consistent with the limit of *k*_obs_ ≥ 0.03 s^-1^ established by the data in Figure S1G.)

Further controls of the assay with Ser102 mutant versions of APs and with R166S AP described below provide additional evidence that the results of the assay accurately reflect P_i_ binding.
