# Supplementary material for: Ground State Destabilization by Anionic Nucleophiles Contributes to the Activity of Phosphoryl Transfer Enzymes
Source: PLoS Biol. 2013 Jul 2;11(7):e1001599. doi: 10.1371/journal.pbio.1001599 (PMC3699461; doi:10.1371/journal.pbio.1001599)
Supplement: Text S3 — Equilibrium-binding assay results with S102G and S102A AP. (DOC) [file pbio.1001599.s022.doc]

**Text S3. Equilibrium-binding assay results with S102G and S102A AP**

The equilibrium-binding assay was used to obtain limits for the P_i_ binding affinity of the AP Ser102 mutants S102G and S102A. We first sought to determine the timescale needed to achieve equilibrium binding. The observed rate constant for binding equilibration equals *k*_on_[AP] + *k*_off_ [13] so we sought to determine *k*_off_. After incubation of the mutant proteins at a concentration shown to result in nearly 100% ^32^P_i_ bound, a chase assay was conducted by adding excess unlabeled P_i_, such that any ^32^P_i_ that dissociates would be replaced by unlabeled P_i_. No significant dissociation of ^32^P_i_ from S102G or S102A AP was observed even after approximately 100 hours (Figure S2A). This result is in contrast to the rapid dissociation observed for WT AP (Figure S1F). These results suggest that the dissociation of P_i_ from S102G or S102A AP (*k*_off_) is slower than 2×10^-7^ s^-1^.

We next measured the uptake kinetics of ^32^P_i_ by these AP mutants. P_i_-free S102G or S102A AP (0.05-10 μM of S102G AP; 0.15-0.4 μM of S102A AP) was combined with ~200 pM ^32^P_i_ and the fraction ^32^P_i_ bound was measured over time. The observed increases in fraction ^32^P_i_ bound at various protein concentrations were fit to yield *k*_obs_ values and the endpoint fraction ^32^P_i_ bound at each protein concentration (Figure S2B and C for S102G and S102A AP, respectively). Given the extremely slow dissociation of ^32^P_i_ indicated by the chase assays described above, it was expected that for each protein concentration used in these uptake assays, the endpoint fraction ^32^P_i_ bound would plateau at the maximum fraction bound as long as the rate constant for binding, *k*_on_, were greater than ~4 M^-1^s^-1^ [e.g., if ≤ 2×10^-7^ s^-1^ and *k*_on_ ≥ 4 M^-1^s^-1^ then the dissociation constant (*K*_d_ = *k*_off_/*k*_on_) would be ≤0.05 μM]. Fits to the observed uptake rate constant, *k*_obs_, versus the [AP] have slopes that give apparent *k*_on_ values, with values of 1300 and 1.6×10^4^ M^-1^s^-1^ for S102G and S102A AP, respectively (Figure S2D and E). However, even though these values are greater than 4 M^-1^s^-1^, the endpoint amounts of bound ^32^P_i_ plateau well below the maximum achievable fraction ^32^P_i_ bound.

To investigate this limited uptake of P_i_, 1 μM of S102G AP that had completed ^32^P_i_ uptake at 0.85 fraction ^32^P_i_ bound was diluted several times to reach a final S102G AP concentration of 0.125 μM. This concentration in the uptake assay (Figure S2B) resulted in less than half of the ^32^P_i_ bound, but the results from the chase assay predict that reducing the S102G AP concentration by dilution would not significantly decrease the fraction ^32^P_i_ bound because, once bound, ^32^P_i_ does not dissociate over this timeframe (Figure S2A). Re-equilibration to lower fraction ^32^P_i_ bound did not occur upon dilution (Figure S3), consistent with the expectations based on the chase assay and suggesting a complexity associated with uptake.

A general model to account for the unexpectedly low fraction ^32^P_i_ binding during the uptake assay is that the amount of free protein capable of binding ^32^P_i_ decreases over the assay time, due to denaturing events, as depicted by the model in Figure S4A. We carried out simulations and nonlinear regression fitting using the KinTek Explorer simulation program [15,16], allowing both a reversible ^32^P_i_ binding equilibrium and an irreversible loss of free, bindable protein according to the scheme of Figure S4A. The *k*_off_ value was fixed as the limiting value from the chase assay. A global fit of this model to the uptake data for all of the S102G AP concentrations (Figure S4B) indicates that a model with irreversible inactivation of free AP but not AP•P_i_ can account for the reduced P_i_ binding at the lower protein concentrations. Values of ~1000 M^-1^s^-1^ and ~3×10^-4^ s^-1^ for *k*_on_ and *k*_inactive_ for S102G AP are obtained. The *k*_on_ value was used in combination with the limit for from the chase assay of ≤2×10^-7^ s^-1^ to provide a limit for the dissociation constant for P_i_ binding (*K*_d_ = /*k*_on_) of ≲0.2 nM (as reported in Table S1 and Table 1 in the main text). This affinity is ≳1000-fold higher than the WT AP affinity with Ser102 intact (; Table 1 and Table S1).

The model of Figure S4A did not yield a good fit to the uptake data for the S102A AP mutant (Figure S4C), particularly at the lower concentrations of S102A AP, presumably due to a less predictable loss of activity. We therefore used the initial uptake data points for S102G and S102A to estimate the *k*_on_ value before the potential protein inactivation process would significantly contribute to the observed uptake. As shown in Figure S5, the initial rates of ^32^P_i_ uptake were estimated by fitting the initial data points at each protein concentration to a line. These initial uptake rate constants were plotted versus the concentration of protein to yield second-order rate constants reflecting the initial uptake. For S102G this was ~700 M^-1^s^-1^ (Figure S5B), in reasonable agreement with the association rate constant estimated from the global fit of the model in Figure S4A (~1000 M^-1^s^-1^). The initial uptake value estimated for S102A was ~1×10^4^ M^-1^s^-1^ (Figure S5D), suggesting that *k*_on_ for this process is ~10-fold faster compared to that for S102G AP. Using this value for *k*_on_ in combination with the limit for , yields a crude estimated limit for the *K*_d_ of P_i_ binding by S102A AP of ≲0.02 nM (Table 1 and Table S1).

Replicate binding assays for S102G and S102A AP showed unexpected high variability in the fraction ^32^P_i_ bound at the endpoint (Figure S6), whereas the other AP variants with weaker binding and faster equilibration gave highly reproducible results (e.g. Figure S1A for WT AP; Figure S8A for S102G/R166S AP; and Figure S8E for S102A/R166S AP) and the kinetics of P_i_ uptake and dissociation for S102G/R166S and S102A/R166S AP agree well with equilibrium-binding measurements (see below and Table S1). The complications for S102G and S102A AP presumably result from an inactivation process that is not fully reproducible. The lack of P_i_ dissociation observed with S102G and S102A AP indicates very strong binding and we turned to S102G/R166S and S102A/R166S AP for quantitative analysis, as the behavior of these mutants was reproducible and self-consistent.
