# Supplementary material for: Ground State Destabilization by Anionic Nucleophiles Contributes to the Activity of Phosphoryl Transfer Enzymes
Source: PLoS Biol. 2013 Jul 2;11(7):e1001599. doi: 10.1371/journal.pbio.1001599 (PMC3699461; doi:10.1371/journal.pbio.1001599)
Supplement: Text S4 — Equilibrium binding of Pi to R166S, S102G/R166S, and S102A/R166S AP. (DOC) [file pbio.1001599.s023.doc]

**Text S4. Equilibrium binding of P_i_ to R166S, S102G/R166S, and S102A/R166S AP**

We determined the P_i_ binding affinity of the R166S, S102G/R166S, and S102A/R166S AP variants. Previous measurements of the R166S AP mutant P_i_ affinity by kinetic inhibition gave *K*_i_ values of 400-500 μM at pH 8.0 [8,17], and we obtained a value of *K*_d_ = 360 μM using this kinetic assay (Figure S7A), in reasonable agreement with the prior measurements. Attempts at measuring the R166S AP affinity by the equilibrium-binding method developed here resulted in a significant decrease in flow rate during filtration with concentrations of R166S AP above 25 μM, suggesting that the filter membrane may become partially blocked with protein. As expected for this mutant, no significant binding was observed over the lower R166S AP concentrations that were accessible in this assay, and the slightly higher than expected binding at 25 μM may have arisen from nonspecific effects related to the above-noted protein blockage (Figure 7B).

We were able to use the equilibrium-binding assay to measure the P_i_ affinities of S102G/R166S and S102A/R166S AP, and this assay was required given the absence of detectable activity of these mutants (Text S1). The fraction ^32^P_i_ bound to each mutant after incubation times in which the fraction ^32^P_i_ no longer changed was plotted against the concentration of protein to generate binding curves with dissociation constants for P_i_ binding of 66 and 77 nM for S102G/R166S and S102A/R166S AP, respectively at pH 8.0 (Figure S8A and E; Table 1; Table S1). Repeat measurements showed very good reproducibility (Figure S8A and E), in contrast to the high variability observed with S102G and S102A AP (Figure S6).

As an additional control, measurement of the kinetics of P_i_ binding gave a calculated dissociation constant (*K*_d_ = *k*_off_/*k*_on_) that was similar to the dissociation constant measured with the equilibrium-binding assay. The dissociation rate constant for each mutant was measured using the chase assay described above (Figure S8B and F). Fits to the observed decrease in the fraction ^32^P_i_ bound (see Methods) yielded values of 1.2×10^-4^ and 1.6×10^-6^ s^-1^ for S102G/R166S and S102A/R166S AP, respectively. The time-dependent uptake of ^32^P_i_ was also measured for these mutants. Fits to the uptake yielded *k*_obs_ values and the maximal fraction of ^32^P_i_ bound (Figure S8C and G). The uptake data fit well to a two-state binding model in which *k*_obs_ = *k*_on_[AP] + *k*_off_ (AP+^32^P_i_AP•^32^P_i_), (Figure S8D and H), yielding *k*_on_ of 1400 and 36 M^-1^s^-1^ for S102G/R166S and S102A/R166S AP, respectively. The y-intercept of these fits, which in principle reflect the dissociation rate constant (*k*_off_), has a high uncertainty because small changes in the fit slope result in relatively large changes to the y-intercept value. Despite the fit error, the y-intercept values from the uptake assay were in reasonable agreement (within 2-4 fold for S102G/R166S and S102A/R166S AP, respectively) with the *k*_off_ values measured more accurately with the chase assay above.

Dissociation constants calculated from the kinetic rate constants and from the fraction ^32^P_i_ bound at endpoints were within 2-fold of one another (Table S1), consistent with simple two-state binding such that *K*_d_ equals *k*_off_/*k*_on_.

The values are several orders of magnitude below the expected value for a diffusion-limited process (10^7^-10^8^ M^-1^s^-1^; *k*_on_ for WT AP is ~1×10^7^ [8,10]), as also suggested by the limited data for S102G and S102A AP above. Slow binding to some proteins has been attributed to slow dissociation of water (or ions) from the binding site [18-24], and the high density of positively charged residues, enhanced by removal of the Ser102 anion, may result in a site that is particularly recalcitrant to exchange of solvent out of the active site.
