# Supplementary material for: Ground State Destabilization by Anionic Nucleophiles Contributes to the Activity of Phosphoryl Transfer Enzymes
Source: PLoS Biol. 2013 Jul 2;11(7):e1001599. doi: 10.1371/journal.pbio.1001599 (PMC3699461; doi:10.1371/journal.pbio.1001599)
Supplement: Text S5 — Interplay between Ser102 and Arg166 revealed by structural comparison. (DOC) [file pbio.1001599.s024.doc]

**Text S5. Interplay between Ser102 and Arg166 revealed by structural comparison**

When Ser102 and Arg166 are both present (WT AP), P_i_ is positioned in a binding mode akin to that of the vanadyl transition state analog. When Arg166 is mutated to serine (R166S AP), the bound P_i_ is rotated and the phosphorus center is translated 1.0 Å relative to its position in WT AP (Figure 3C) [8]. One model to account for the P_i_ binding mode in R166S AP is that in the absence of Arg166, Ser102 displaces P_i_ from the preferred binding mode. In these structures, Ser102 is likely protonated, but its presence may nevertheless have some destabilizing influence on bound P_i_ as suggested by the structural comparison in Figure 3C. This model accounts for the observation that removal of Ser102 from R166S AP (to give S102G/R166S AP) allows bound P_i_ to return to the WT position, akin to the vanadyl transition state analog position.

The above structural comparison suggests that Arg166 is needed to position P_i_ when Ser102 is present. As the P_i_ position is very similar to that of the vanadyl transition state analog (Figure 3A), this conclusion is consistent with a previous energetic analysis indicating that Arg166 plays a role in specific transition state stabilization [8]. The new observation that Arg166 is not needed to position P_i_ when Ser102 is absent (Figure 3D) provides structural support for a destabilizing ground state effect from the Ser102 nucleophile.
