# Supplementary material for: Ground State Destabilization by Anionic Nucleophiles Contributes to the Activity of Phosphoryl Transfer Enzymes
Source: PLoS Biol. 2013 Jul 2;11(7):e1001599. doi: 10.1371/journal.pbio.1001599 (PMC3699461; doi:10.1371/journal.pbio.1001599)
Supplement: Text S6 — Estimation of affinity for AP with Ser102 deprotonated. (DOC) [file pbio.1001599.s025.doc]

**Text S6. Estimation of PO affinity for AP with Ser102 deprotonated**

The PO affinity for deprotonated Ser102 AP can be estimated by measuring the P_i_ affinity across a pH range. In principle, the observed P_i_ binding at a given pH can reflect the binding contributions of any of the P_i_ species. The results presented in the main text indicate that the observed P_i_ affinity in the neutral pH range for R166S AP (and for WT AP as shown in Figure S9C and published previously [6,7]) reflects the formal binding of HPO. The binding of free HPO is presumably accompanied by a net internal proton transfer to give neutral Ser102 and bound PO as shown by the equilibrium defined by in Figure S9A. As the pH approaches the p*K*_a_ of HPO (11.7), the proportion of PO in solution increases and the overall observed P_i_ affinity can start to reflect a direct binding contribution from PO, if that affinity is sufficiently strong ( in Figure S9A); an increase in the observed P_i_ affinity as the pH is raised would indicate a binding contribution from PO. The size of this contribution will depend on the solution pH and the PO affinity relative to the HPO affinity. As all of the Ser102 AP will be deprotonated at these pH values (p*K*_a_ ≤ 5.5; [6]), PO binding that increases as pH increases would be to AP with Ser102 deprotonated.

The observed pH-dependent P_i_ binding to AP is complicated by an inactivating p*K*_a_ (p*K*) associated with free AP. Nevertheless, this inactivating titration can be accounted for (using pH-dependent tungstate binding measurements as described in the main text) and if PO makes a binding contribution at higher pH values, an upward trend is expected. To ensure that AP remains functional even at high pH values, *p*NPP hydrolysis activity throughout the pH range was measured, and as *p*NPP has no titratable protons in this pH range it was expected that a continuous log-linear decrease in activity reflecting only p*K* would be observed. A continuous log-linear decrease in activity was observed to pH 11.4 for WT AP [6,7] and to pH 10 for R166S AP (Figure S9B).

The P_i_ affinity data from pH 7.0-11.4 for WT AP and pH 6-10 for R166S AP are shown in Figure S9C and D, respectively. No upward-trend from PO binding was detected. Thus, we could set a lower limit for . To estimate this value, Equation S3, derived from the model in Figure S9A, was used to fit the pH-dependent data with and p*K* fixed based on the fits assuming HPO binding only. The PO affinity in Equation S3 was fixed at a series of decreasing values (Figure S9C and D). Clear deviations from the high pH data are observed if is set to values lower than 100 nM for WT AP and lower than 2.5 μM for R166S AP, so these values give conservative lower limits (i.e., ≥ 100 nM and 2.5 µM for WT and R166S AP, respectively; Table 2).
