# Supplementary material for: Ground State Destabilization by Anionic Nucleophiles Contributes to the Activity of Phosphoryl Transfer Enzymes
Source: PLoS Biol. 2013 Jul 2;11(7):e1001599. doi: 10.1371/journal.pbio.1001599 (PMC3699461; doi:10.1371/journal.pbio.1001599)
Supplement: Text S7 — 31P NMR of the R166S AP•Pi complex suggests bound . (DOC) [file pbio.1001599.s026.doc]

**Text S7. ^31^P NMR of the R166S AP•P_i_ complex suggests bound PO**

Vibrational spectroscopy of P_i_ associated with WT AP allowed assignment of PO as the bound species [7]. Analogous experiments with R166S AP were not successful because of low signal-to-noise even at high enzyme concentrations (3.3 mM). We therefore could not use vibrational spectroscopy to assign the P_i_ species bound to R166S AP as was done previously for WT AP [7]. We instead assigned the P_i_ species bound to R166S AP by comparing the ^31^P NMR spectra of R166S and WT AP, as follows.

The ^31^P NMR spectrum of P_i_ with WT AP at pH 8.0 shows a chemical shift of ~3.7 ppm (Figure S10A) (see also [11,26,27]). Based on the vibrational data noted above, this P_i_ species was assigned as PO [7]. If PO were also bound to R166S AP, a similar ^31^P NMR chemical shift for the bound P_i_ would be expected in the simplest case. The chemical shift observed for P_i_ in the presence of R166S AP (Figure S10A) varies depending on the fraction of P_i_ that is bound (*K*_d_ value of 360 μM at pH 8.0; Figure S7A). The exchange of R166S AP-bound and unbound P_i_ is fast, estimated as ~6×10^4^ s^-1^ (= *k*_exchange_ = *k*_on_[R166S AP] + *k*_off_ = (3.3×10^7^ M^-1^s^-1^)(1.3 mM) + 1.5×10^4^ s^-1^; [8]), relative to the NMR timescale, which suggests that the observed single peak is a population-averaged peak of unbound and bound P_i_. [The rate constant of P_i_ exchange needed to observe a population-averaged peak can be estimated from the chemical shift difference between bound (3.8 ppm, *vida infra*) and unbound (2.19 ppm, Table S3) P_i_ and the ^31^P frequency (162 MHz) of the NMR spectrometer. These parameters correspond to a frequency difference of 260 Hz, above which discrete peaks for unbound and bound P_i_ would not be resolved. The exchange value of 6×10^4^ s^-1^ estimated above is much greater than 260 s^-1^, consistent with the observation of a single peak.] As 100% P_i_ binding could not be readily achieved at accessible concentrations of protein, we determined the observed chemical shift versus the fraction P_i_ bound and extrapolated to estimate the chemical shift of fully bound P_i_ (Figure S10B). This extrapolated chemical shift is ~3.8 ppm, within error of the chemical shift of P_i_ bound to WT AP. The result suggests that the same P_i_ species are bound to both WT and R166S AP.
