# Supplementary material for: Ground State Destabilization by Anionic Nucleophiles Contributes to the Activity of Phosphoryl Transfer Enzymes
Source: PLoS Biol. 2013 Jul 2;11(7):e1001599. doi: 10.1371/journal.pbio.1001599 (PMC3699461; doi:10.1371/journal.pbio.1001599)
Supplement: Text S8 — 31P NMR of Pi-bound to S102G and S102G/R166S AP with excess Pi. (DOC) [file pbio.1001599.s027.doc]

**Text S8. ^31^P NMR of P_i_-bound to S102G and S102G/R166S AP with excess P_i_**

^31^P NMR was used to probe whether multiple P_i_ species bind to S102G/R166S AP. Previous results with WT AP showed that the chemical shift of noncovalently bound P_i_ remains constant over a pH range from 5-10 [7], consistent with vibrational data that also suggest that a single P_i_ species binds over this pH range [7]. Similarly, the chemical shift of S102G-bound P_i_ does not change with pH (Figure S11A), suggesting that a single P_i_ species binds across this pH range. The data in the main text provide strong evidence that this species is PO (see “pH-dependent P_i_ binding to AP without the Ser102 nucleophile” in Results and Discussion). In contrast, the ^31^P NMR chemical shift observed for P_i_ in the presence of S102G/R166S AP varies systematically at different pH values (Figure 6). The single, variable chemical shift could reflect pH-dependent binding of multiple P_i_-bound forms that are in rapid exchange or a rapid exchange process between bound and unbound P_i_.

We distinguished between these models as follows. If the observed chemical shift reflects multiple bound P_i_ forms then addition of excess P_i_ would result in the appearance of a new peak, reflecting the added, unbound P_i_. In contrast, if the observed chemical shift reflects rapid exchange binding of bound and unbound P_i_ then addition of excess P_i_ would result in a shift of the original peak toward the expected chemical shift value of unbound P_i_, without the appearance of a new peak. Addition of P_i_ in excess of the S102G/R166S AP concentration resulted in the appearance of a new ^31^P peak at each pH, as expected for the former model with multiple bound P_i_ forms. One peak had a chemical shift corresponding to the expected unbound P_i_ shift at that pH (see Table S3) and the other with a chemical shift corresponding to that for the sample without excess P_i_ (Figure S11B). The chemical shift that is observed both in the absence and presence of excess P_i_ is not perturbed by the presence of free P_i_, strongly suggesting that this chemical shift represents that of P_i_ bound to S102G/R166S AP in multiple forms whose relative populations depend on the pH of the solution.
