# Supplementary material for: Ground State Destabilization by Anionic Nucleophiles Contributes to the Activity of Phosphoryl Transfer Enzymes
Source: PLoS Biol. 2013 Jul 2;11(7):e1001599. doi: 10.1371/journal.pbio.1001599 (PMC3699461; doi:10.1371/journal.pbio.1001599)
Supplement: Text S9 — Equations derived from the models in Figure 4C and D to fit the pH-dependent Pi binding data for R166S (Equation S1) and S102G/R166S (Equation S2) AP in Figure 4A. (DOC) [file pbio.1001599.s028.doc]

**Text S9. Equations derived from the models in Figure 4C and D to fit the pH-dependent P_i_ binding data for R166S (Equation S1) and S102G/R166S (Equation S2) AP in Figure 4A.**

(Equation S1)

(Equation S2)

The model in Figure 4D from which Equation S2 is derived contains a thermodynamic cycle between the solution HPO and PO species, the binding affinities of each species, and the S102G/R166S AP-bound equilibrium between HPO and PO. From the ^31^P NMR measurements in Figure 6, the S102G/R166S AP-bound equilibrium constant () is 10^-6.1^ M, and this value was used as a constraint in obtaining the fit of the pH-dependent data in Figure 4A from Equation S2 as follows. The relationship of the thermodynamic cycle in Figure 4D gives ×= ×. Rearranging yields ()/() = ()/() and ()/() = (10^-6.1^ M)/(10^-11.7^ M) = 10^5.6^, and this ratio was held constant in the fit to the data in Figure 4A.
