# Supplementary material for: Ground State Destabilization by Anionic Nucleophiles Contributes to the Activity of Phosphoryl Transfer Enzymes
Source: PLoS Biol. 2013 Jul 2;11(7):e1001599. doi: 10.1371/journal.pbio.1001599 (PMC3699461; doi:10.1371/journal.pbio.1001599)
Supplement: Text S10 — Comparison of AP•Pi affinities with AP Ser102 protonated, deprotonated, or mutated to Gly. (DOC) [file pbio.1001599.s029.doc]

**Text S10. Comparison of AP•P_i_ affinities with AP Ser102 protonated, deprotonated, or mutated to Gly**

Most simply, it was expected that the PO affinities of AP with Ser102 removed by mutation or with Ser102 neutralized by protonatation would be approximately equal because both result in the removal of the proposed destabilizing anion and electrostatic repulsion. The PO affinity of AP with protonated Ser102 was computed using a thermodynamic cycle (Figure 5A) involving the observed P_i_ affinity at pH 8.0, the HPO p*K*_a_, and the upper limit of the Ser102 p*K*_a_. Because the Ser102 p*K*_a_ is an upper limit (≤5.5; [6]), the PO dissociation constant values calculated using the thermodynamic cycle are upper limits, with of ≤69 pM and ≤290 fM for R166S and WT AP, respectively (Table 2 and Figure S13; grey bars). Assuming the simplest model, that the Ser102 p*K*_a_ is 5.5, gives affinities in the same range as those for the S102G mutants (Figure S13; cf. black and grey bars) but the PO affinity of AP with Ser102 protonated is somewhat lower (~10^2^-fold) than the PO affinity of AP with Ser102 mutated. The structural comparisons of WT and variant APs in Figure 3 show that Ser102, which is likely protonated in these structures, displaces P_i_ when Arg166 is not present to buttress P_i_ in the preferred WT binding mode. This observation suggests that protonated Ser102 may retain some fractional destabilization effect on P_i_ binding. Alternatively, if the Ser102 p*K*_a_ is actually lower, ~3.5 instead of 5.5, then the calculated PO affinity of AP with Ser102 protonated would be the same as the PO affinity of AP with Ser102 mutated.
