# Supplementary material for: Ground State Destabilization by Anionic Nucleophiles Contributes to the Activity of Phosphoryl Transfer Enzymes
Source: PLoS Biol. 2013 Jul 2;11(7):e1001599. doi: 10.1371/journal.pbio.1001599 (PMC3699461; doi:10.1371/journal.pbio.1001599)
Supplement: Text S11 — Previous estimation for the destabilization from Ser102 on the binding of a dianionic phosphate. (DOC) [file pbio.1001599.s030.doc]

**Text S11. Previous estimation for the destabilization from Ser102 on the binding of a dianionic phosphate**

A previous estimate of the dianionic substrate destabilization from Ser102 was obtained by first comparing the dissociation constants for PO binding to WT AP with Ser102 deprotonated (see Figure S9C; *K* ≥ 100 nM) and to WT AP with Ser102 neutralized by protonation (see Figure 5; ≤ 290 fM) yielding a destabilization of ≥3.4×10^5^-fold, which corresponds to a free energy difference of ≥7.5 kcal/mol [ΔΔG = *RT*ln(*K*/)] [7]. To estimate the destabilization from Ser102 on dianion binding it was assumed that the destabilization from Ser102 on PO trianion binding scaled with the charge difference between a trianion and a dianion, such that the dianion destabilization was calculated to be two-thirds of the trianion destabilization in energetic terms [≥5 kcal/mol = (-2/-3)×(≥7.5 kcal/mol)]; this value would correspond to a binding destabilization for a dianion of ≥4.8×10^3^-fold. This value assumed that the destabilization from Ser102 scales log-linearly with the charge of the P_i_ ligand, but this relationship need not hold. Nevertheless, the current work shows that the Ser102 destablization of trianion binding is ≥10^8^-fold and that the destabilization of dianion binding is ≥10^3^-fold, similar to the prior estimate. In energetic terms, trianion binding is destabilized by ≥10.9 kcal/mol [= *RT*ln(≥10^8^)] and dianion binding is destabilized by ≥4.1 kcal/mol [= *RT*ln(≥10^3^)]. Because these values are both limits we cannot determine the difference in the Ser102 destabilization for dianion versus trianion binding.
