# Supplementary material for: Ground State Destabilization by Anionic Nucleophiles Contributes to the Activity of Phosphoryl Transfer Enzymes
Source: PLoS Biol. 2013 Jul 2;11(7):e1001599. doi: 10.1371/journal.pbio.1001599 (PMC3699461; doi:10.1371/journal.pbio.1001599)
Supplement: Text S12 — Estimation of the contribution of Arg166 to binding of Pi dianion. (DOC) [file pbio.1001599.s031.doc]

**Text S12. Estimation of the contribution of Arg166 to binding of P_i_ dianion**

The dissociation constant for HPO binding the S102G AP mutant with Arg166 present is very likely lower than the dissociation constant determined for HPO binding the S102G/R166S AP mutant (= 90 nM; Table 2) due to favorable contacts between the nonbridging oxygen atoms of HPO and Arg166. However, the very strong binding of PO by S102G AP with K_d_ ~ 1 fM masked our ability to measure HPO binding. We conservatively estimate that Arg166 contributes an additional ~10-fold to the binding of HPO based on 1) its previously measured contribution to stabilization of the dianionic phosphoserine intermediate (E-P species) of 90-fold [8] and 2) structural evidence showing Arg166 in position to hydrogen bond with the oxygen atoms of bound HPO [29,30]. In the simplest scenario, we expect HPO to bind in the presumed substrate binding orientation with a proton in place of the leaving group monoester portion of the substrate, as illustrated in Figure 7. In this orientation, Arg166 can make hydrogen bonds to both nonbridging oxygen atoms of the bound dianion that are deprotonated. However, the HPO proton could occupy one of the phosphate oxygen atoms that is expected to hydrogen bond to Arg166. In this scenario we still expect Arg166 to contribute to binding because functional studies show that Arg166 contributes ~40-fold to the catalysis of methyl-*p*NPP hydrolysis in which the methyl group is attached to one of the phosphate oxygen atoms that would ordinarily contact Arg166 [9]. This result suggests that Arg166 can make a significant binding contribution of at least 10-fold even if the bound ligand disrupts one of the hydrogen bonds from Arg166.
