# Supplementary material for: Ground State Destabilization by Anionic Nucleophiles Contributes to the Activity of Phosphoryl Transfer Enzymes
Source: PLoS Biol. 2013 Jul 2;11(7):e1001599. doi: 10.1371/journal.pbio.1001599 (PMC3699461; doi:10.1371/journal.pbio.1001599)
Supplement: Text S13 — Comparison of and binding by S102G AP. (DOC) [file pbio.1001599.s032.doc]

**Text S13. Comparison of HPO and PO binding by S102G AP**

The binding results and analysis in the main text provide estimates for the strong binding of both HPO and PO by S102G/R166S and S102G AP but with much stronger binding of PO. We have measured this differential affinity for S102G/R166S as 4.3×10^5^-fold (Table 2; / for S102G/R166S AP), and a somewhat larger differential affinity of ~10^7^-fold is expected with Arg166 present in the S102G AP mutant (Table 2; / for S102G AP). Presumably, PO binds more strongly than HPO because each of the three anionic oxygen atoms of PO has more charge to interact with the positively charged AP active site residues (-0.75 vs. -0.67 formal charge per oxygen for PO and HPO, respectively). In addition, the fourth oxygen atom of HPO is protonated, has no formal charge, and likely makes weaker electrostatic interactions relative to the analogous oxygen atom of PO, although it is not known which oxygen atom of the dianion is protonated. The large binding affinity preference of AP for the more negatively charged PO shown here is consistent with previous work with AP that showed a very steep increase in catalytic activity for substrates with increasing negative charge [31].
