# Supplementary material for: Ground State Destabilization by Anionic Nucleophiles Contributes to the Activity of Phosphoryl Transfer Enzymes
Source: PLoS Biol. 2013 Jul 2;11(7):e1001599. doi: 10.1371/journal.pbio.1001599 (PMC3699461; doi:10.1371/journal.pbio.1001599)
Supplement: Text S14 — Evidence against electrostatic repulsion in phosphoryl transfer transition states. (DOC) [file pbio.1001599.s033.doc]

**Text S14. Evidence against electrostatic repulsion in phosphoryl transfer transition states**

Because of the extreme resilience of phosphate esters to hydrolysis, early on it was proposed that electrostatic repulsion between the incoming nucleophile and the phosphate ester in the transition state hinders the reaction [33,34]. However, two subsequent studies bear directly on this question and provide evidence that electrostatic repulsion from formal charge on the incoming nucleophile does not significantly hinder phosphoryl transfer. The reactivity of both neutral and negatively charged oxygen nucleophiles with negatively charged phosphorylated 4-methyl pyridine fall on the same correlation line versus nucleophile p*K*_a_ [35-37], providing evidence against significant electrostatic repulsion in the transition state for the oxyanion reactions. In addition, increasing the ionic strength had only a 5-fold differential effect on the reaction rates of an oxyanion nucleophile versus a neutral nucleophile [36], providing no indication of strong electrostatic repulsion in the transition state.
