# Supplementary material for: Ground State Destabilization by Anionic Nucleophiles Contributes to the Activity of Phosphoryl Transfer Enzymes
Source: PLoS Biol. 2013 Jul 2;11(7):e1001599. doi: 10.1371/journal.pbio.1001599 (PMC3699461; doi:10.1371/journal.pbio.1001599)
Supplement: Text S15 — Extended implications for phosphoryl transfer catalysis. (DOC) [file pbio.1001599.s034.doc]

**Text S15. Extended implications for phosphoryl transfer catalysis**

As noted in the main text, the destabilization identified from Ser102 is just one of several components that imparts catalysis in AP. One of the other more obvious catalytic contributions, other than the destabilization discussed in the main text, is the positioning of the Ser102 nucleophile with respect to the reactive phosphoryl group, and as noted above, this can be viewed as transition state stabilization as well as destabilization of the E•S complex relative to freely diffusing nucleophile and substrate in solution. While we have no quantitative information on the magnitude of this catalytic contribution in AP, a previous study that used imidazole to chemically rescue activity of a kinase lacking its histidine nucleophile suggests that a contribution from nucleophile positioning of at least ~10^2^-fold is possible for phosphoryl transfer [38].

Another catalytic contribution in AP very likely arises from the interaction between Zn^2+^ and the leaving group oxygen atom of the phosphoryl group, as a large buildup of negative charge on this oxygen in the transition state is expected (Figure 7; [4,6,14]). Also, additional preferential stabilization may arise from geometrical differences between the tetrahedral ground state and the trigonal bipyramidal transition state that allow stronger interactions between AP active site residues and the nonbridging oxygen atoms of the transferred phosphoryl group. Indeed, previous measurements indicate that interactions between the nonbridging oxygen atoms of the phosphoryl group and Arg166 strengthen by ~10-fold compared to the corresponding interactions in the tetrahedral ground state [8].

Our results suggest that ground state destabilization from anionic nucleophiles plays an important role in catalysis. But not all phosphoryl transfer enzymes use anionic nucleophiles, raising the question of how these enzymes achieve sufficient ground and transition state discrimination without the proposed ≳10^3^-fold contribution from anionic nucleophile ground state destabilization. For example, many ATPases have been proposed to use a neutral water nucleophile (typically aided by an enzymatic base to remove a proton during or after the chemical step; for review see [39-42]). The uncatalyzed hydrolysis of the nucleoside triphosphate substrate is much faster than the uncatalyzed hydrolysis of phosphate monoesters (*k*_uncat_ ~ 5×10^-11^ M^-1^s^-1^ for GTP and ATP hydrolysis vs. *k*_uncat_ ~ 4×10^-22^ M^-1^s^-1^ for methyl phosphate hydrolysis at 25 °C [40,43,44]) so that a much smaller rate enhancement is needed by ATPases than phosphatases. Thus, ATPases and other phosphoryl transfer enzymes that utilize activated substrates may not require all types of potential catalytic contributions and mechanisms for ground state discrimination. Interestingly, enzymes with NTP substrates may employ ground state destabilization by orienting the bound γ and β phosphoryl groups of the nucleotide substrate in an eclipsed position, thereby inducing electrostatic repulsion that is subsequently relieved as the γ phosphoryl group moves away from the β phosphoryl group in the transition state [45-48].

Histidine phosphatases [49] and kinases [50] also use a neutral nucleophile, histidine, that presumably cannot make use of ground state destabilization via electrostatic repulsion from the active site nucleophile but still achieve high rate enhancements, similar to their anionic nucleophile-containing enzyme counterparts. The ~10^2^-fold stronger intrinsic reactivity of nitrogen nucleophiles than oxygen nucleophiles (i.e., a preference for transition state formation) with phosphate compounds may compensate for a missing contribution from ground state destabilization [51]. It is also possible that the lone pair electrons of the formally neutral nucleophile imparts some destabilization. Future comparative mechanistic analyses will help reveal commonalities and unique mechanisms employed by the many enzymes that catalyze the phosphoryl transfer reactions that are central to biology.
